# Changes in high-frequency aperiodic 1/f slope and periodic activity reflect post-stimulus functional inhibition in the visual cortex

**DOI:** 10.1162/imag_a_00146

**Published:** 2024-04-26

**Authors:** Viktoriya O. Manyukhina, Andrey O. Prokofyev, Tatiana S. Obukhova, Tatiana A. Stroganova, Elena V. Orekhova

**Affiliations:** Center for Neurocognitive Research (MEG Center), Moscow State University of Psychology and Education, Moscow, Russian Federation; National Research University Higher School of Economics, Moscow, Russian Federation

**Keywords:** magnetoencephalography, aperiodic 1/f slope, post-stimulus functional inhibition, alpha power, beta power, alpha frequency, visual cortex

## Abstract

It has been shown that cessation of intensive sensory stimulation is associated with a transient increase in functional inhibition in the sensory cortical areas. However, the electrophysiological correlates of this post-stimulus inhibition in the human brain have not been thoroughly investigated. To investigate post-stimulus inhibition, we analyzed magnetoencephalogram (MEG) recorded at rest and after cessation of visual stimulation of varying intensity (high-contrast gratings drifting at a slow, medium, or high rate) in 25 healthy women aged 18–40 years. We analyzed condition- and intensity-related changes in MEG parameters sensitive to functional inhibition: periodic alpha-beta power, peak alpha frequency (PAF), and 1/f aperiodic slope. We also investigated the association of these parameters with sensory sensitivity and avoidance assessed by a questionnaire. To evaluate the influence of hormonal status on the studied parameters, participants were examined twice, during the follicular and luteal phases of the menstrual cycle (MC). Regardless of the MC phase, increasing drift rate of visual gratings resulted in a proportional increase of post-stimulus posterior alpha-beta power, PAF, and a negative shift of the aperiodic (1/f) slope of the power spectrum in the high-frequency range. Compared to rest, the post-stimulus periods were characterized by higher PAF, more negative 1/f slope in posterior cortical areas, and a widespread increase in beta power. While condition- and drift-rate-dependent modulations of alpha-beta power and 1/f slope were correlated, changes in PAF did not correlate with either of them. A greater intensity-dependent increase in visual alpha-beta power predicted higher subjective sensory sensitivity/avoidance, suggesting stronger regulatory top-down modulation of the visual cortex in individuals with heightened sensitivity. Our results show that several MEG parameters concordantly indicate a post-stimulus enhancement of inhibition that is proportional to the intensity of the preceding visual stimulation. While post-stimulus changes in alpha-beta power and 1/f slope may share some common mechanisms, changes in PAF reflect a distinct aspect of inhibitory regulation. Our results inform potential inhibition-based biomarkers for clinical and translational research.

## 1 Introduction

A balance between neural excitation and inhibition (E/I balance) in neural circuits is crucial for the normal functioning of the brain and its disruption is associated with a variety of neuropsychiatric disorders (Anticevic & Murray, 2017;Sohal & Rubenstein, 2019). Recent advances in understanding the role of E/I balance in the etiology of these disorders instigated a search for its non-invasive and accessible biomarkers that could help to stratify patients according to the dominant neural deficit and be used in clinical and translational research.

Several classes of non-invasive electrophysiological biomarkers of the E/I balance have been suggested which are usually based on electroencephalographic (EEG) and magnetoencephalographic (MEG) parameters estimated “at rest” or during sensory stimulation (seeAhmad et al., 2022for review). Yet, the neural activity recorded*after cessation*of intensive sensory stimulation may provide additional valuable information regarding the link between MEG/EEG parameters and regulation of the E/I balance. According to cellular (Shmuel et al., 2006;Q. Zhang et al., 2023), blood-oxygen-level dependent (BOLD) functional magnetic resonance imaging (fMRI) (Havlicek et al., 2015;Mullinger et al., 2013;Sadaghiani et al., 2009), MEG (Stevenson et al., 2011), and EEG (Mullinger et al., 2013,^2017^) studies, the period after stimulation is dominated by inhibition, that is, suppression of excitatory neuronal activity. Since the post-stimulus neural suppression may originate from multiple sources, followingMayhew et al. (2022)andIemi et al. (2022)here we will use the term post-stimulus inhibition (or functional inhibition) to refer to a decrease in the E/I ratio that can occur via activation of GABAergic inhibitory interneurons and/or via decreased excitatory drive (e.g., downregulation of norepinephrine and acetylcholine).

In the primate visual cortex, termination of visual input is immediately followed by local decreases of multiunit neural activity below the baseline level (Logothetis et al., 2001). In human fMRI, a cessation of visual stimulation is accompanied by the “undershoot”—a negative (i.e., below pre-stimulus baseline) BOLD response that at least in part is accounted for by the shift of the E/I ratio to inhibition (Havlicek et al., 2015;Mullinger et al., 2013). Recent studies have shown that fMRI BOLD undershoot is reduced in elderly people (Mayhew et al., 2022) and in people with some neuropsychiatric disorders (Havlicek et al., 2015;Murray et al., 2020), suggesting weakened neural inhibition and altered E/I ratio.

Whereas the hemodynamic undershoot in humans has been extensively investigated (J. J. Chen & Pike, 2009;Haigh et al., 2015;Mullinger et al., 2013,^2017^;Stevenson et al., 2011), less attention has been paid to the MEG/EEG indices of post-stimulus inhibition. Since MEG and EEG directly reflect neural functioning, they may appear even more informative than the BOLD fluctuations regarding the regulation of E/I balance in healthy and diseased human brain.

In EEG and MEG, the cessation of visual stimulation is followed by an increase in alpha and beta power in the visual cortex (Mullinger et al., 2017;Stevenson et al., 2011). Similarly to fMRI BOLD undershoot, the post-stimulus alpha-beta synchronization differs from that observed during baseline, and in this case, it*exceeds*the pre-stimulus baseline level (Mullinger et al., 2017;Stevenson et al., 2011). Importantly, this post-stimulus alpha “overshoot” correlates with the fMRI undershoot on a trial-by-trial basis (Mullinger et al., 2017), which suggests that both phenomena reflect inhibition associated with the termination of visual stimulation. Indeed, increases in periodic alpha and beta power have been previously associated with reduced excitability as evidenced by their negative correlations with high-frequency broadband gamma activity (Iemi et al., 2022) and neuronal spiking (Chapeton et al., 2019;Dougherty et al., 2017;Haegens et al., 2011).

The frequency of alpha oscillations, although not investigated in the context of post-stimulus inhibition, has also been shown to reflect state-related changes in cortical excitability. For example, the increased frequency of posterior alpha oscillations during the retention period of a memory task is thought to reflect inhibition of sensory processing, because it is accompanied by slower reaction time and decreased neural responses to external stimuli (Babu Henry Samuel et al., 2018). Furthermore, the spontaneous fluctuations of alpha frequency are inversely associated with BOLD activity in the visual cortex (Babu Henry Samuel et al., 2018). Given these findings, we hypothesized that, along with changes in alpha and beta power, enhanced inhibition after strong visual stimulation would be associated with an acceleration of alpha oscillations.

Another promising but unexplored indicator of the E/I ratio after the cessation of sensory stimulation is the slope of the aperiodic 1/f-like part of the power spectrum. The aperiodic slope is steeper during “down states” such as sleep and anesthesia compared to wakefulness (Colombo et al., 2019;Muthukumaraswamy & Liley, 2018), during wakefulness with “eyes closed” compared to “eyes open” (Manyukhina, Prokofyev, et al., 2022;F. Zhang et al., 2019), and during awake states characterized by decreased information processing compared to more active states (Ouyang et al., 2020;Pietrelli et al., 2022). The steepness of the aperiodic spectral slope is thought to reflect the neural E/I ratio so that a flatter (less negative) slope is associated with a higher background neuronal firing rate, decoupled from the oscillatory carrier frequency, that is, with higher “noise” (Gao et al., 2017;Voytek et al., 2015). Given that neuronal firing rate is a hallmark of the E/I ratio in neuronal networks, a steeper (more negative) aperiodic slope in the post-stimulus interval compared to the resting state may be a more direct indicator of decreased excitability than power of periodic alpha-beta oscillations.

To be consistent with the functional inhibition hypothesis, the neurophysiological indices should change in proportion to the strength of the respective sensory input. Indeed, fMRI BOLD and near-infrared spectroscopy (NIRS) oxyhemoglobin undershoots in the visual cortex scale with an intensity of the preceding stimulation (Haigh et al., 2015;Mullinger et al., 2013;Sadaghiani et al., 2009;Stevenson et al., 2011). At the same time, information regarding the influence of the strength of the preceding stimulation on the spatial and spectral features of post-stimulus MEG and EEG is scarce and inconsistent. Stevenson et al analyzed beta MEG power and found greater “beta rebound” after the presentation of drifting versus static grating in the visual area MT, but not in the primary visual cortex (Stevenson et al., 2011).Mullinger et al. (2017), on the other hand, observed a significantly greater increase in EEG alpha power after flickering than after static visual stimulus, but they did not localize the sources of this “alpha rebound” effect.

In the present study, we analyzed MEG recorded during time intervals that followed visual stimulation of varying intensity, as well as during passive visual fixation (“rest”). We pursued several complementary goals.

*First,*we investigated how various MEG parameters, which had previously been associated with functional inhibition, change after cessation of visual stimulation as compared to the “rest”. Apart from the periodic alpha and beta power, we estimated peak alpha frequency (PAF) and the slope of the 1/f part of the power spectrum. There is evidence that the choice of the frequency range for estimating the aperiodic 1/f slope can strongly affect the results (e.g.,Manyukhina, Orekhova, et al., 2022;Maschke et al., 2023;Ossandón et al., 2023). A recent MEG study shows that aperiodic part of the MEG spectrum is best described by a model with two power laws and a knee at approximately 15 Hz (Ibarra Chaoul & Siegel, 2021). Two distinct aperiodic slopes may also be present in the EEG, as evidenced by oppositely directed changes in aperiodic slopes measured at low (<~15–20 Hz) and high (>~15–20 Hz) frequencies after ketamine administration (Colombo et al., 2019) or as a result of visual deprivation (Ossandón et al., 2023). As animal and modeling studies have linked the E/I ratio to an aperiodic slope in the high-frequency region (around 30–50 Hz;Gao et al., 2017), in the present study we estimated the MEG spectral slope in the range of 35–45 Hz, the same approach we used in a previous study in children (Manyukhina, Prokofyev, et al., 2022). We have chosen this narrow frequency range to avoid spontaneous periodic oscillations, which are normally absent at frequencies higher than ~30 Hz (Lendner et al., 2020) and to minimize the contribution of high-frequency ambient noise to the aperiodic slope (Ibarra Chaoul & Siegel, 2021).

*Second*, we sought to find out whether these MEG indices of functional inhibition are proportional to the intensity of the preceding visual stimulation. To modulate the intensity of visual stimulation, we varied the drift rate of a high-contrast visual grating that moved toward the center of the visual field. We have previously shown that increasing gratings’ velocity effectively modulates the excitatory state of the visual cortex during the stimulation period, as reflected in changes in high-frequency gamma oscillations (Orekhova et al., 2019,^2020^) and in the magnitude of pupil constriction (Orekhova et al., 2018).

*Third*, we explored the correlations between the inhibition-sensitive MEG parameters. Although these parameters have been extensively studied individually, the relationship between them is poorly understood. At the same time, the phenomenon of post-stimulus functional inhibition can provide information about this relationship and help elucidate its underlying functional mechanisms.

Our*fourth*aim was to test whether differences in MEG indices of post-stimulus inhibition reflect individual differences in sensory perception. Since visual hypersensitivity is associated with heightened excitability of the visual cortex (Bargary et al., 2015), it may also manifest in an altered post-stimulus inhibition. We considered two possibilities. First, more sensitive individuals may have generally weaker inhibition, which would lead to weaker post-stimulus changes in inhibition-sensitive MEG parameters. Indeed, sensory hypersensitivity in Fragile X Syndrome (FRAX) (Ethridge et al., 2017;Razak et al., 2021) and autism (Q. Chen et al., 2020) is thought to reflect a global GABAergic inhibitory deficit. Second, hypersensitive healthy individuals may compensate for increased excitability by downregulating the excitatory activity of the stimulated sensory areas through inhibitory top-down connections. In line with this hypothesis, it has been shown that subjects with somatic anxiety and elevated sensory sensitivity have increased backward inhibitory effective connectivity in exteroceptive sensory networks (Bouziane et al., 2022). Therefore, if the post-stimulus inhibition is under top-down control, it may be even stronger in hypersensitive individuals. To explore the putative relationship between post-stimulus MEG activity and individual differences in sensory processing, we assessed in our subjects “Low Neurological Threshold” using the Sensory Profile questionnaire (Brown & Dunn, 2002) and investigated whether subjects’ scores on this measure could be predicted by MEG measures of post-stimulus inhibition.

The experimental sample consisted of women who participated in our previous studies devoted to the effect of the menstrual cycle (MC) on visual gamma oscillations (Manyukhina, Orekhova, et al., 2022;Manyukhina et al., 2021). Whereas in the previous studies we investigated the direct effect of visual stimulation intensity during stimulus presentation, in the present study we analyzed the time intervals*after*the cessation of these visual stimuli as well as the resting state. The participants were investigated twice, during the follicular and luteal phases of the MC, which provided us with additional information on the role of women’s hormonal status and allowed us to evaluate the between-phase consistency of the metrics used.

## 2 Methods

### 2.1 Participants

Twenty-five healthy females from 18 to 40 years of age (27.9 ± 6.0 years) with normal or corrected vision were recruited for an experiment among university students or among participants of the “healthy-lifestyle” internet community aimed at learning healthy habits and reducing risk factors for health problems. Exclusion criteria were the presence of a psychiatric disorder, smoking, irregular MC, and treatment with hormonal therapy. These subjects also participated in our previous study of the effect of MC on brain oscillations (Manyukhina, Orekhova, et al., 2022). Therefore, for each individual, MEG data were collected twice, during the follicular and luteal phases, with an interval between recordings from 7 to 147 days. The experimental data analyzed in the current study were obtained during these visits. However, unlike the earlier study, where we examined oscillatory responses to visual stimulation, here we focused on the resting state and post-stimulus changes that have not been previously analyzed.

The investigations were conducted in accordance with the Declaration of Helsinki and were approved by the Ethical Committee of the Moscow State University of Psychology and Education. All participants provided verbal assent to participate in the study and were informed about their right to withdraw from the study at any time during testing. They also gave written informed consent after the experimental procedures had been fully explained.

### 2.2 Adolescent/adult sensory profile

The participants were asked to fill in the Russian version of the Adolescent/Adult Sensory Profile (A/ASP) (Brown & Dunn, 2002). The A/ASP questionnaire assesses the subject’s sensory processing in daily life according to Brown & Dunn’s four quadrants model: “Sensory Sensitivity”, “Low Registration”, “Sensory Seeking”, and “Sensory Avoidance”. In addition, A/ASP allows the assessment of a Low Neurological Threshold, which is calculated as the sum of the Sensory Sensitivity and Sensory Avoidance scales and measures a person’s notice of or annoyance with sensory stimuli of different modalities. Here, to reduce the number of correlations, we used Low Neurological Threshold as an integral measure of subjects’ sensory sensitivity and avoidance and tested how scores on this scale correlate with the MEG indices of post-stimulus inhibition.

### 2.3 MEG experimental design

**Fig. 1.**
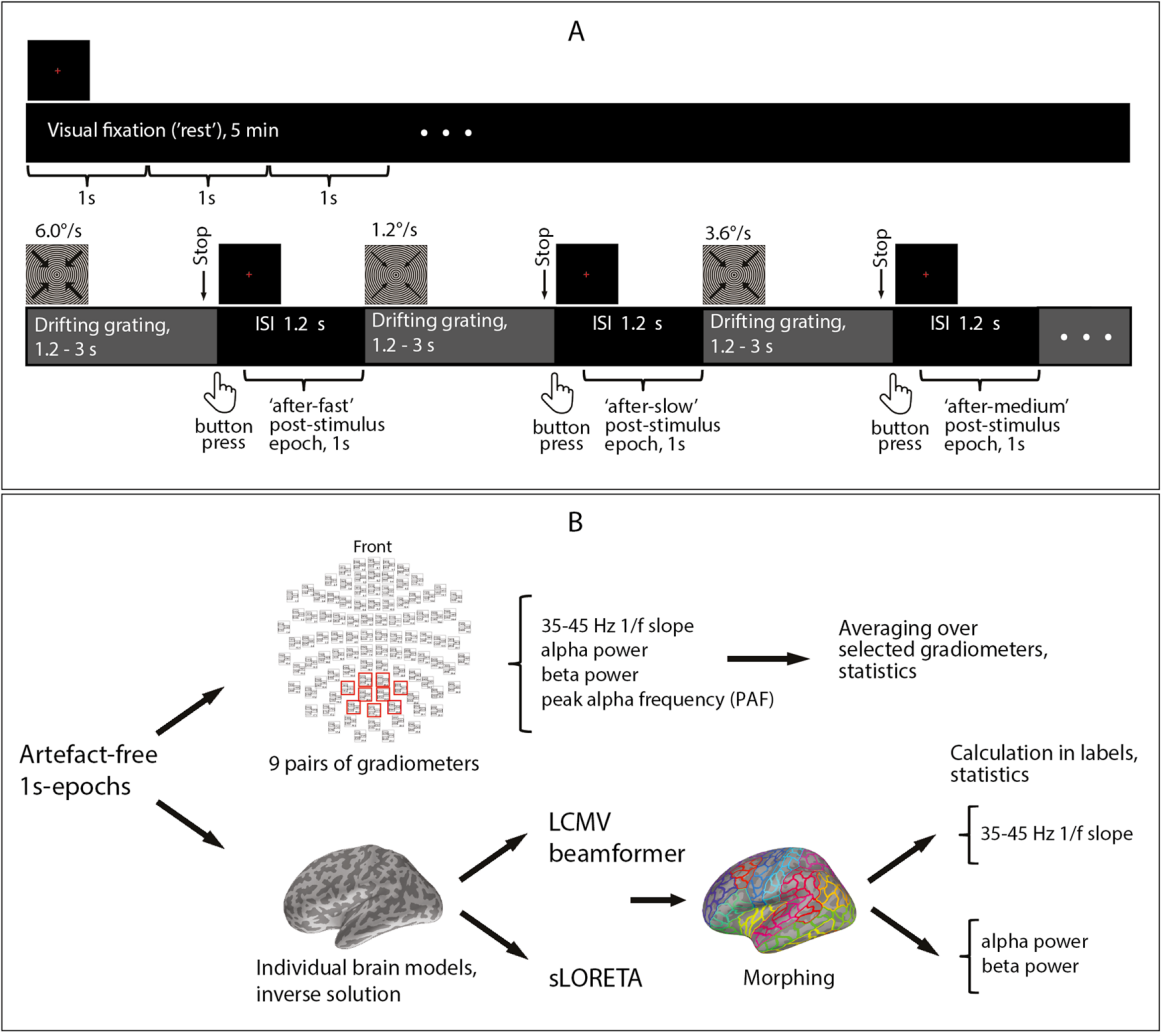
Schematic representation of experimental design and analysis pipeline. (A) MEG was recorded at rest and during a visual task; 1-s epochs after stimulus cessation (marked by brackets) were used for the analysis. (B) Analysis was first performed in the “sensor space”, where several MEG parameters were calculated for the selection of posterior gradiometers. Changes in these parameters (except PAF, see Methods) that showed a significant effect of Condition (rest vs. post-stimulus intervals) or of Intensity (“after-slow” vs. “after-fast” post-stimulus intervals) were localized in the brain.

MEG was recorded under two experimental conditions, at rest and during a visual task (Fig. 1A). During rest, subjects fixated their gaze on a small red cross in the center of the screen for 5 min. The visual task is described in detail in our previous studies (Orekhova et al., 2018,^2019^). In short, the participants were presented with large (18° of visual angle) high-contrast circular grating that remained static (0°/s) or moved with one of three velocities: 1.2°/s (“slow”), 3.6°/s (“medium”), and 6.0°/s (“fast”) for 1.2–3 s. Each trial started with a fixation cross which lasted for 1.2 s and was followed by the presentation of a grating of one of the four types in random order. The participants were asked to respond to stimulation changes (motion of initially static grating or cessation of motion of the moving grating) by pressing a button with the right or left index finger (counterbalanced), after which the fixation cross appeared and the next trial began. As the instruction was slightly different for the static and moving stimuli, for the purpose of the present study we only analyzed intervals following moving gratings. The number of omission and commission errors (>1000 ms and <150 ms relative to the target event, respectively) was low (mean = 4.1%, std = 2.7%), suggesting that participants attended to the task most of the time. In total, 90 gratings of each type were presented to each individual during three sessions interrupted by short breaks. Short cartoon movies were presented after each 2–5 grating stimuli in order to maintain the subject’s attention and decrease boredom.Figure 1Ashows schematic representation of the experimental design.

### 2.4 MEG data acquisition and preprocessing

MEG data were acquired with an Electa VectorView Neuromag 306-channel MEG detector array (Helsinki, Finland) consisting of 102 magnetometers and 204 planar gradiometers at the Moscow Center for Neurocognitive Research (MEG-center).

Data were registered with 0.03 Hz high-pass and 300 Hz low-pass inbuilt filters and sampled at 1000 Hz. Temporal signal-space separation method (tSSS) (Taulu & Hari, 2009) from MaxFilter software (v.2.2) was applied to the raw MEG signal to reduce external noise and compensate for head movements.

Next preprocessing steps were performed using MNE-python software (v.0.24) (Gramfort et al., 2013). They included data down-sampling at 500 Hz and correction for biological artifacts (heartbeats and vertical eye movements) using Independent Component Analysis (ICA). From 1 to 4 ICs were excluded in each recording (mean ± standard deviation (SD): 2.32 ± 0.68 and 2.04 ± 0.34 for the rest and visual task, respectively). The raw data were then filtered at 1–45 Hz to analyze periodic alpha, beta power, and at 25–145 Hz to analyze the aperiodic 1/f slope in the 35–45 Hz range (see below). For the rest condition, the raw data were divided into 1 s non-overlapping epochs. For the visual task, epochs of 1-s duration were extracted, spanning the period from 0.2 to 1.2 s after cessation of visual stimulation/appearance of the fixation cross (i.e., post-stimulus intervals). Epochs of both types were visually inspected based on unfiltered epoched data, and those contaminated by muscle artifacts or high-amplitude noise were excluded from the analysis. Post-stimulus epochs were further divided according to the type of preceding stimulation (drift rate 1.2°/s, 3.6°/s, or 6.0°/s) into three categories: “after-slow”, “after-medium”, and “after-fast”, respectively. Post-stimulus epochs preceded by cartoon movies were excluded from the analysis. The final number of the clean post-stimulus epochs was 58.76 ± 6.86, 58.04 ± 6.71, and 56.74 ± 6.96 (mean ± SD) for the “after-slow”, “after-medium”, and “after-fast” conditions, respectively. The number of clean epochs for the rest condition exceeded that for the visual task (all drift velocities combined). To equalize the number of epochs, for each subject, we took the number of clean rest epochs equal to the number of clean post-stimulus epochs from the beginning of the rest condition. For further analysis, we used data from 204 planar gradiometers.

### 2.5 MRI data acquisition and processing

Structural MRI data were acquired in all the participants (General Electric Signa 1.5 Tesla MRI scanner; voxel size 1 mm  ×  1 mm  ×  1 mm). T1-weighted anatomical images were processed using the standard “recon-all” pipeline implemented in FreeSurfer software (v.6.0.0) (Fischl et al., 2002) which runs all the default preprocessing steps. For the localization of effects in the brain, the cortical surface was parceled into 448 similar-size labels using anatomical atlas byKhan et al. (2018).

### 2.6 Estimation of aperiodic slopes, alpha and beta power, and peak alpha frequency (PAF) in sensor space

For the sensor space analysis, all MEG data were translated to a standard position (0, 0, 40). Since our visual stimuli induce the strongest neural response in posterior gradiometers (Fig. 1BinOrekhova et al., 2015), we expected the post-stimulus inhibition to be strongest in the same locations. Therefore, for the analysis at the sensor level we selected nine pairs of gradiometers, as shown inFigure 1B. We did not include sensors located close to the edges of the MEG helmet in this selection because their signal is frequently contaminated by cranial and neck muscle activity.

To calculate PSD, we applied Welch’s method (“psd_welch” function in MNE-Python software; from 1 to 50 Hz, frequency resolution = 0.1 Hz, with zero padding, no overlap) on individual 1-s epochs. The resulting spectra were averaged over epochs and selected gradiometers, separately for the two visits and four conditions: rest and three types of post-stimulus intervals (Fig. 1A; seeFig. S1inSupplementary Materials 1andSupplementary Materials 2for individual and grand average spectra in each visit and condition). The averaged PSD were further used to estimate the aperiodic 1/f slopes in 35–45 Hz ranges, periodic alpha and beta powers and PAF at the sensor level.

To estimate the 1/f slope, PSD values were interpolated using interpolate.interp1d function from Scipy library in Python v.3.7 in order to have an equal distance between frequency bins on a logarithmic scale between 35–45 Hz. We then fitted the linear function into the logarithm of the PSD versus the logarithm of frequency using “polyfit” function (Python library NumPy, v.1.21.4), the same as we did inManyukhina, Prokofyev, et al. (2022)(Supplementary Materials 1,Fig. S2).

To extract periodic component, we applied FOOOF (Fitting Oscillations & One-Over-F) method. Previously,Ibarra Chaoul and Siegel (2021), while using another popular approach to parameterization of neural power spectrum—IRASA (Irregular Resampling Auto-Spectral Analysis), reported that the model with two power laws and noise term best described the MEG data (Wen & Liu, 2016). IRASA could not be used in our study because the post-stimulus epochs were too short. Our attempt to use the “FOOOF” algorithm (Donoghue et al., 2020) with two power laws over a wide frequency range (2–40 Hz) led to reasonably good fits (all R^2^> 0.94), but to a large variation in knee frequencies between subjects and conditions (1.8 to 26.9 Hz) that was physiologically unlikely. Therefore, to separate the aperiodic and periodic components in the 2–40 Hz range, we fitted the FOOOF model with a “fixed” mode (i.e., no “knee”) for the “aperiodic_mode” parameter. All other parameters were set to default (peak_width_limits = (0.5,12), max_n_peaks = inf, min_peak_height = 0, peak_threshold = 2).

The periodic component of the power spectrum was estimated as:

$$\begin{array}{l} \text{Periodic }=\text{ Original spectrum }\\ \;\;\;\;\;\;\;\;\;\;\;\;\;\;\;\;\;\;\;\;\;\;\;\;\;\;\;\;\;\;\;-\text{ Spectrum without periodic peaks}, \end{array}$$

where “Spectrum without periodic peaks” was calculated using model._spectrum_peak_rm FOOOF function.

The FOOOF model without the “knee” provided a good explanation for the data (R^2^> 0.96 for all subjects and conditions), but the absolute error of the fit increased at slow (2 Hz) and high (>25 Hz) frequencies (Supplementary Materials 1,Fig. S3). The error was, however, lower in the alpha-beta range (8–25 Hz) and did not differ in this range between rest and post-stimulus condition (Student’s t = 0.8, df = 24, p = 0.43), or between three types of post-stimulus intervals (rmANOVA: F(2,48) = 0.45, p = 0.64). The spectral slope estimated with this method differed neither between rest and post-stimulus period (F(1,24) = 0.05, p = 0.83) nor between the three post-stimulus intervals following different stimulation intensities (rmANOVA: F(2,48) = 0.51, p = 0.6). This suggested that when periodic activity was separated from aperiodic component using the single-slope FOOOF model, its modulation by experimental conditions could be reasonably accurately estimated. The periodic component was then used to calculate mean alpha and beta power.

It has been shown that the MEG alpha rhythm may operate at higher frequencies than the canonical upper alpha bound of 12 Hz (Capilla et al., 2022;Haegens et al., 2014) that is particularly characteristic of experimental conditions associated with functional loads (Haegens et al., 2014). Indeed, visual inspection of occipital spectra revealed in some participants clear dominant peaks at around 13–14 Hz during the post-stimulus period, although at rest in the same participants the alpha peak fell within the “conventional” frequency range of 8–12 Hz. Therefore, we used the extended alpha range of 8–14.3 Hz in this study. The beta band of 15–25 Hz was used to provide some separation between the functional alpha and beta activity.

To distinguish alpha frequency changes from changes in alpha-beta power, we estimated the frequency of the highest peak in the alpha band (i.e., peak alpha frequency, PAF) rather than the weighted alpha frequency. The peak in PSD was identified using “signal.find_peaks” function from Scipy library (v.1.7.3). As the alpha peak was present in all the conditions only in 22 subjects, PAF was calculated in 22 of 25 women.

### 2.7 Source space analysis

For each visit session and for each condition, the data were corrected to the subject’s “optimal” initial head position (seeOrekhova et al., 2023for details). Individual structural MRIs were co-registered with the subject’s MEG recordings using “mne_analyze” tool from MNE-C software (https://mne.tools/stable/install/mne_c.html). Further steps of source-level analysis were performed using MNE-python software (v.0.24) (Gramfort et al., 2013). For each subject, a single-layer boundary element model and surface-based source space with 4098 vertices in each hemisphere were created, and a forward solution was estimated.

To estimate the periodic alpha and beta power, the raw data were filtered at 1–45 Hz, and standardized low-resolution brain electromagnetic tomography (sLORETA) inverse solution (Pascual-Marqui, 2002) was computed. For the analysis of aperiodic slope, the raw data were filtered at 25–145 Hz. In the latter case, to solve the inverse problem we used the linearly constrained minimum variance (LCMV) beamformer. The choice in favor of different inverse solution methods was made based on their different advantages and disadvantages. LCMV beamformer, together with the broad-band filtration in the high-frequency range, is preferable for analysis of aperiodic slope in the high-frequency range because it enables better filtering of noise and myogenic artifacts than sLORETA (Manyukhina, Prokofyev, et al., 2022). sLORETA, on the other hand, does not have depth bias in the source localization and therefore provides more realistic cortical localization of spectral power than LCMV beamformer (Tait et al., 2021).

Filtered raw data were epoched, and bad epochs (seeSection 2.4) were discarded. Different spatial filters were calculated for the two types of comparisons. To compare post-stimulus intervals that followed different types of stimulation (“after-slow”, “after-medium”, “after-fast”), the common spatial filter was calculated for these three conditions. For the comparison between rest and post-stimulus intervals pooled across stimulus types, a common spatial filter was calculated for all of these data. For the source reconstruction with the LCMV beamformer, the noise covariance parameter in the filter estimation function was set to “None”, which was possible because the analysis was performed for one type of sensors (gradiometers). The unit-gain LCMV beamformer filter was created with the orientation that maximizes power, regularization 0.1, and the “reduce_rank” parameter set to “True”. For sLORETA, noise covariance was estimated from the empty room MEG data, which were preprocessed in the same way as the raw data. “Loose” and “depth” parameters were set to 0.4 and 0.8, respectively. The LCMV beamformer filter or the sLORETA inverse operator was applied to each epoch individually.

MEG parameters in the source space were estimated in the same way as those in the sensor space, but in this case, PSD was calculated at the level of vertex sources and averaged within each of 448 cortical labels chosen according to an anatomical parcellation (Khan et al., 2018). To estimate the aperiodic slope, the PSD at each individual vertex was normalized to its maximal value before averaging. This was done to ensure that all vertices contributed equally to the average. We did not analyze PAF in the source space, because many participants lacked distinct alpha peaks beyond parieto-occipital cortical areas.

### 2.8 Statistical analysis

To estimate the reliability of the inhibition-sensitive MEG variables between phases of the MC, we calculated intraclass correlation coefficients (ICCs). ICCs were estimated for a fixed set of “raters” (follicular and luteal MC phases) (Koo & Li, 2017).

For the sensor-level analysis, all variables were checked for deviation from a normal distribution using Shapiro-Wilk’s W test. Alpha and beta spectral power values were preliminary log10-transformed to normalize the distributions. The resulting distributions did not differ significantly from the Gaussian. Levene’s test was used to test for homogeneity of variance. As none of the analysis of variance (ANOVA) assumptions was violated, we then used repeated-measures ANOVAs (rmANOVAs) to test for the effects of Condition*or*Intensity and Phase (follicular, luteal), as well as for the interaction effects. Factor Condition had two levels (rest and post-stimulus period pooled across all trials), while the factor Intensity had three levels (“after-slow”, “after-medium”, and “after-fast”). When appropriate, the Greenhouse-Geisser correction was applied to correct for violation of the sphericity assumption.

For the source-space analysis, in each of the 448 cortical labels, we used Student’s t-test for related samples to test for the difference between rest and post-stimulus condition or between post-stimulus intervals following cessation of the most and least intensive stimulation (i.e., “after-fast” vs. “after-slow” intervals). To control for multiple comparisons, we applied Benjamini-Hochberg false discovery rate (FDR) correction (Benjamini & Hochberg, 1995) with a threshold of 0.05 to the p-values.

To test for a relationship between condition- or intensity-related changes in MEG parameters and Low Neurological Threshold, we estimated Pearson correlation coefficients. In this case, changes in spectral power between conditions or intensities were quantified in %, as 2*(A-B)/(A+B)*100%, while changes in PAF and aperiodic slopes were calculated as the absolute differences: A–B.

## 3 Results

To investigate the sensitivity of MEG parameters to post-stimulus enhancement of inhibition, we followed two lines of analysis. First, we compared time intervals following cessation of visual stimulation (all three types of post-stimulus epochs combined) with the resting state, when subjects passively fixated their gaze on a central cross for a long period of time (Fig. 1A). We expected to find concordant changes in aperiodic slope, alpha-beta power, and PAF that would indicate enhancement of inhibition during the post-stimulus period compared to rest. Second, to find out whether the inhibition-sensitive MEG parameters change in proportion to the intensity of the preceding stimulation, we compared post-stimulus intervals that followed visual stimulation of increasing intensity: gratings drifting at “slow”, “medium”, and “fast” rates.

We started with an analysis in the “sensor space”. In this case, each of the parameters was averaged over a selection of nine pairs of posterior gradiometers (Fig. 1B) and subjected to rmANOVA to test for the effects of stimulation condition or intensity. In addition, for each parameter, we estimated the ICC between the two phases of the MC. We then analyzed the cortical localization of significant condition- and intensity-related differences found at the sensor level.

### 3.1 Sensor space analysis

#### 3.1.1 Goodness-of-fit of the linear model in the 35-45 Hz range

To estimate goodness-of-fit of the linear model to the log-transformed spectrum in the 35–45 Hz range we calculated normalized root mean square error (nRMSE) as RMSE / (max value–min value). This produces a value between 0 and 1, where values closer to 0 represent better fit. The nRMSE was on average 0.058 and 0.053 for the resting and combined post-stimulus conditions and 0.083, 0.082, and 0.085 for the after-slow, after-medium, and after-fast intervals, respectively, indicating that the model covers, on average, more than 90% of the variability.

To test whether the residual variance differs between the experimental conditions, we compared original (i.e., not normalized) RMSEs. The RMSE did not differ between rest and post-stimulus conditions (Student t = 0.11, df = 24, p = 0.92), or between three types of post-stimulus intervals (rmANOVA: F(2,48) = 0.27, p = 0.77).

#### 3.1.2 Intraclass correlations (ICCs) of the MEG parameters

To find out whether the investigated parameters demonstrated rank-order stability across the two phases of the MC, we calculated ICCs between the two visits (during follicular and luteal phases of the MC). Nearly all ICCs were good (>0.75) (Koo & Li, 2017), suggesting high stability (Table 1). It should be noted that a low ICC does not necessarily indicate low stability, as it may reflect a systematic but individually specific effect of MC.

**Table 1.** ICCs of the MEG parameters between the recording sessions performed on different days (in follicular and luteal phases of MC).

	Post-stimulus time interval following visual motion				Rest
	1.2°/s (“Slow”)	3.6°/s (“Medium”)	6.0°/s (“Fast”)	All intervals combined	
Alpha peak frequency	0.77 (0.54 - 0.90) *	0.81 (0.59 - 0.91)	0.81 (0.60 - 0.92)	0.84 (0.65 - 0.93)	0.85 (0.69 - 0.94)
Alpha power	0.72 (0.47 - 0.87)	0.89 (0.77 - 0.95)	0.89 (0.77 - 0.95)	0.85 (0.70 - 0.93)	0.85 (0.69 - 0.94)
Beta power	0.77 (0.55 - 0.89)	0.86 (0.71 - 0.93)	0.81 (0.62 - 0.91)	0.82 (0.63 - 0.91)	0.82 (0.63 - 0.92)
Aperiodic 1/f slope	0.79 (0.59 - 0.90)	0.83 (0.66 - 0.92)	0.76 (0.54 - 0.89)	0.86 (0.72 - 0.94)	0.85 (0.68 - 0.94)

* 95% confidence intervals are given in parentheses.

#### 3.1.3 The link between aperiodic slope and periodic alpha and beta power

It has been previously shown that a steeper aperiodic slope correlates with stronger periodic alpha activity (Muthukumaraswamy & Liley, 2018;Podvalny et al., 2015). Here, we tested if this correlation with periodic power would be reproduced for 1/f slope estimated in the narrower (35–45 Hz) frequency range. Since both alpha and beta oscillations are associated with a reduction of neuronal activity (Iemi et al., 2022), we tested for correlation of aperiodic slopes with both alpha and beta periodic power. The aperiodic 1/f slope estimated indeed negatively correlated with alpha and beta periodic power during both rest and post-stimulus period, but not with PAF (Table 2).

**Table 2.** Pearson correlations of aperiodic spectral slope (estimated by two different methods) with periodic alpha power, periodic beta power, and peak alpha frequency (PAF).

	Rest			Post-stimulus time interval		
	Periodic alpha power (N = 25)	Periodic beta power (N = 25)	PAF (N = 22)	Periodic alpha power (N = 25)	Periodic beta power (N = 25)	PAF (N = 22)
Aperiodic 1/f slope 35-45 Hz						
*Follicular phase*	-0.66 **	-0.60 **	0.29	-0.68 **	-0.67 **	0.30
*Luteal phase*	-0.62 **	-0.61 **	0.29	-0.66 **	-0.69 **	0.21

** p < 0.004, FDR corrected.

N, number of subjects.

#### 3.1.4 Comparison between rest and post-stimulus period

**Fig. 2.**
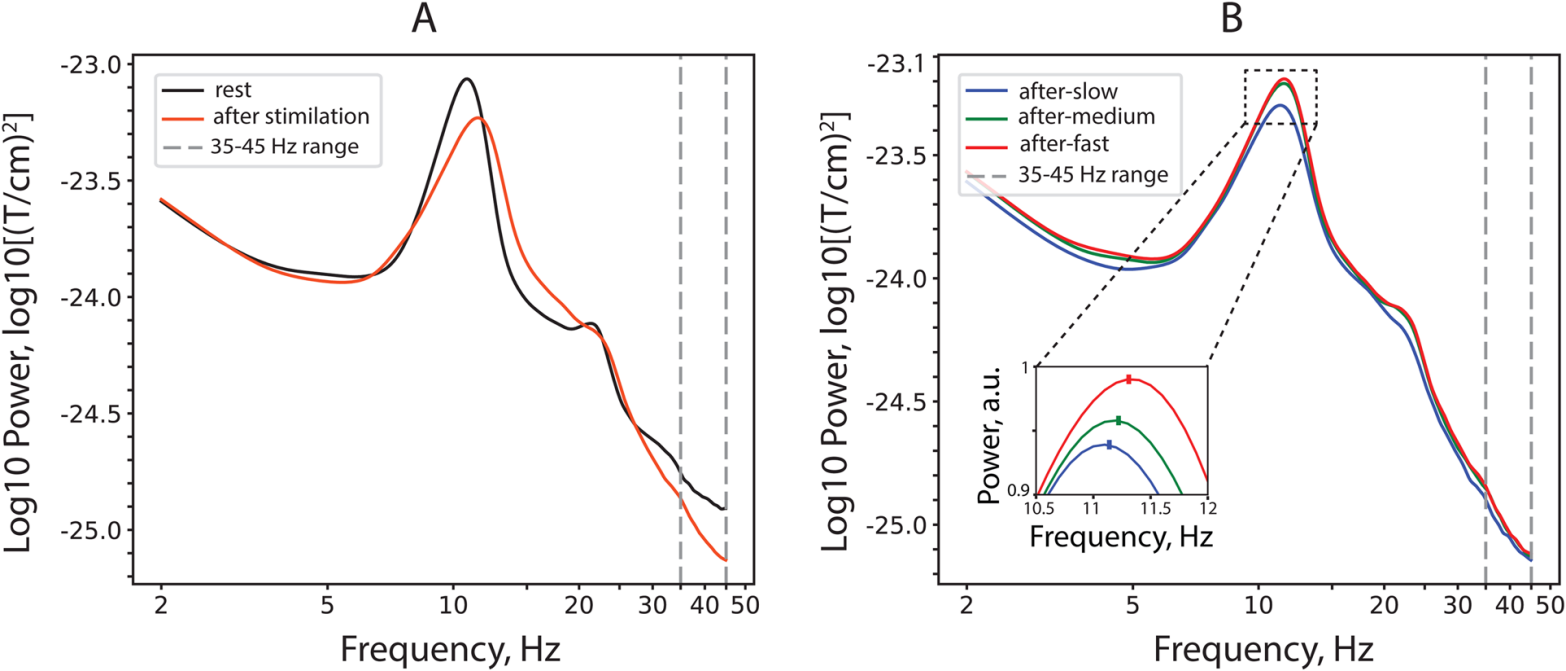
Grand-average log-transformed power spectra for the rest and post-stimulus conditions. (A) Rest and combined post-stimulus intervals. (B) Intervals after cessation of stimulation with slow-, medium-, and fast-drifting gratings. The insert that zooms on the alpha peak shows relative power (i.e., each frequency bin is normalized by the sum over the 2–40 Hz range and multiplied by 100). Only subjects with a distinct alpha peak in all conditions are included (N = 22). Note that post-stimulus PAF (marked with a dash in the insert) increases proportionally to the drift rate of the preceding grating.

Figure 2Ashows the power spectra for the two experimental conditions—rest and period after cessation of visual stimulation averaged for all participants over nine pairs of posterior gradiometers and two visits across all participants.

Comparisons between MEG parameters estimated during rest and post-stimulus intervals are shown inFigure 3A. The rmANOVAs with Condition (rest, post-stimulus) and Phase (follicular, luteal) factors showed that the 1/f slope was steeper during the post-stimulus period (F(1,24) = 13.8, p = 0.001; η_p_^2^= 0.35). We assumed that if the condition-related differences in the 35–45 Hz aperiodic slope in our study are related to muscle artifacts that are typically evident in MEG at frequencies above 20 Hz (Muthukumaraswamy, 2013), then they should be more pronounced at edge gradiometers closer to the neck muscles (Supplementary Materials 1, Results,Fig. S4). However, additional analysis (seeSupplementary Materials 1, Results) revealed no significant effect of Condition (or Intensity) for edge gradiometers, suggesting that the observed effect is unlikely to be explained by between-condition differences in muscle artifacts.

**Fig. 3.**
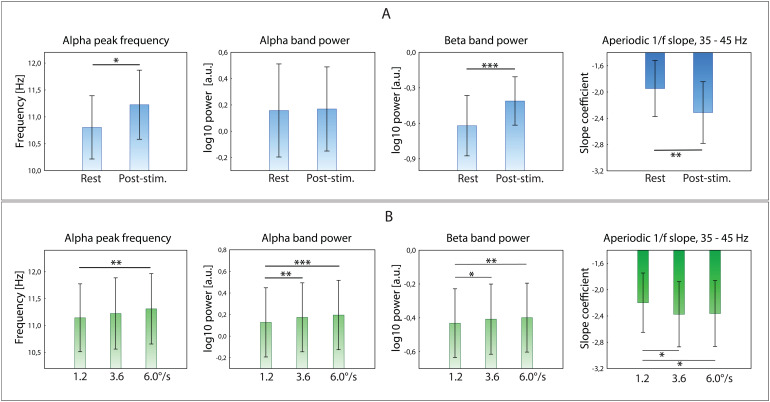
Effects of Condition and stimulation Intensity on MEG indices of functional inhibition. MEG parameters were averaged over nine pairs of posterior gradiometers. (A) Comparison of conditions: rest vs. post-stimulus interval. (B) Comparisons of post-stimulus intervals as a function of preceding stimulation intensity (i.e., grating’s drift rate). *p < 0.05, **p < 0.01, ***p < 0.001.

A spectral alpha peak in all conditions was present in 22 of 25 participants. In these participants, PAF increased during the post-stimulus period relative to rest (Mean_rest_= 10.8, SD = 0.28; Mean_post_= 11.2, SD = 0.31; F(1,21) = 8.0, p = 0.01; η_p_^2^= 0.27).

Periodic beta power strongly increased during the post-stimulus period compared to rest (F(1,24) = 27.9, p = 0.00002; η_p_^2^= 0.54), while no condition-related differences were observed for periodic alpha power (F(1,24) = 0.03, p = 0.87). A lack of alpha power reactivity to a putative increase of inhibition in the ISI might be explained, at least in part, by the counteracting effect of increased post-stimulus inhibition and increased tonic arousal on alpha power. In subjects with a low level of tonic arousal in a calm resting-state condition, a visual task requiring attention can elicit an alerting effect. In such subjects, the level of arousal during post-stimulus task intervals may be higher than during the resting period, resulting in a decrease in alpha power. Consistent with this hypothesis, subjects with higher than average resting alpha power levels (and likely low arousal level in this condition) also showed a significant decrease in alpha power during the post-stimulus period (Wilcoxon signed-rank test: Follicular phase, N = 7, Z = 2.0, p = 0.04; Luteal phase: N = 9, Z = 2.1, p = 0.04), while no such effect was observed in subjects with below-average resting-state alpha power (both p’s > 0.6).

The effect of the MC Phase was significant only for PAF: alpha frequency was higher in the luteal than in the follicular phase (follicular Mean = 10.9, SD = 0.27; luteal Mean = 11.2, SD = 0.29; F(1,21) = 12.9, p = 0.002; η_p_^2^= 0.38).

#### 3.1.5 Effect of the intensity of the preceding stimulation

Figure 3Bshows the comparison of the MEG parameters between time intervals that followed the cessation of gratings drifting at different rates. The rmANOVAs with factors Intensity (“after-slow”, “after-medium”, “after-fast”) and Phase (follicular, luteal) showed a significant main effect of intensity on post-stimulus alpha power (F(2,48) = 15.2, p = 0.00001; η_p_^2^= 0.39), beta power (F(2,48) = 6.9, p = 0.002; η_p_^2^= 0.22), PAF (F(2,42) = 4.5, p = 0.02; η_p_^2^= 0.18), and 1/f aperiodic slope (F(2,48) = 4.1, p = 0.02; η_p_^2^= 0.15). Alpha power, beta power, and PAF increased, while the 35-45 Hz 1/f slope became steeper as the intensity of the preceding stimulation increased.

**Fig. 4.**
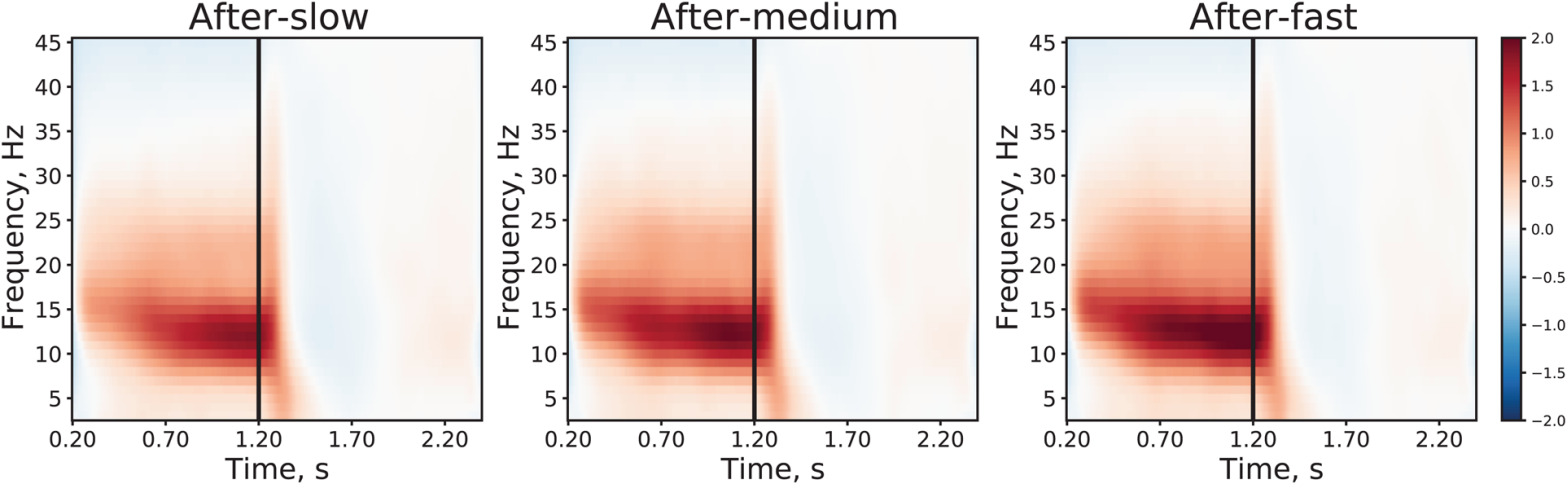
Grand average time-frequency plots showing post-stimulus changes in power after presentation of a visual grating drifting at slow, medium, and fast rates. Power was averaged over subjects, visits and nine pairs of posterior gradiometers, separately for each of the three stimulation conditions. The next stimulation interval (1.5–2.4 s) was used as a baseline. The vertical line at 1.2 s corresponds to the end of the 1.2-s post-stimulus period and the beginning of the next stimulation. Color scale corresponds to (post-stimulus–baseline)/baseline power ratio. Because different stimuli (“slow”, “medium”, or “fast”) were presented in random order, they contributed equally to the baseline interval. Note that increasing in the drift rate of the preceding visual grating results in an increase in alpha and beta band power, rather than a change in the temporal course of the oscillatory response.

Figure 4illustrates the power changes during the post-stimulus intervals in the time-frequency domain. Time-frequency plots showed that increasing the drift rate of the preceding stimulation did not affect the time courses of alpha-beta power, but instead increased power uniformly throughout the post-stimulus interval. Thus, the observed changes in periodic components are compatible with an intensity-dependent increase in post-stimulus functional inhibition.

Again, the effect of Phase was significant only for the PAF (follicular Mean = 11.10, SD = 0.40; luteal Mean = 11.4, SD = 0.39; F(1,21) = 8.5, p = 0.008; η_p_^2^= 0.29). There were no significant Condition x Phase interactions.

#### 3.1.6 Correlations between condition-related changes in MEG indices of functional inhibition

To assess whether changes in the MEG parameters sensitive to inhibition reflect similar underlying mechanisms, we calculated correlations between their changes (Tables 3Aand3B). Increasing steepness (negativity) of 1/f slope from “after-slow” to “after-fast” post-stimulus interval correlated (or tended to correlate) with an increase in alpha-beta power. Changes in PAF did not correlate with changes in other parameters even as a tendency.

**Table 3A.** Correlation of changes in inhibition-sensitive MEG parameters between rest and post-stimulus period.

	Peak alpha frequency Post-stimulus – Rest	Periodic alpha power Post-stimulus/Rest	Periodic beta power Post-stimulus/Rest
Periodic alpha power Post-stimulus/Rest	-0.13 (N = 22)		
Periodic beta power Post-stimulus/Rest	-0.11 (N = 22)	0.56 * (N = 25)	
1/f slope 35–45 Hz Post-stimulus–Rest	-0.10 (N = 22)	-0.36 (N = 25)	-0.41# (N = 25)

* p < 0.05, FDR corrected. Here and hereafter, the parameters were averaged over two visits.

**Table 3B.** Correlation of changes in inhibition-sensitive MEG parameters between “after-slow” and “after-fast” conditions.

	Peak alpha frequency “after-fast” – “after-slow”	Periodic alpha power “after-fast”/“after-slow”	Periodic beta power “after-fast”/“after-slow”
Periodic alpha power “after-fast”/“after-slow”	0.06 (N = 22)		
Periodic beta power “after-fast”/“after-slow”	-0.34 (N = 22)	0.76 *** (N = 25)	
1/f slope 35–45 Hz “after-fast”–“after-slow”	0.07 (N = 22)	-0.40 # (N = 25)	-0.48 * (N = 25)

#p < 0.1, *p < 0.05, ***p < 0.001, FDR corrected.

To sum up, the sensor-level analysis suggested that the MEG parameters putatively sensitive to functional inhibition changed from rest to post-stimulus condition and scaled with an intensity of preceding stimulation. The increase of post-stimulus inhibition as compared to rest was manifested by an increase in beta power, alpha frequency, and the steepening of the 1/f aperiodic slope estimated in the 35–45 Hz frequency range. The scaling of post-stimulus inhibition with the increase of the preceding stimulation intensity was reflected in concordant changes in the four investigated parameters: increase of alpha and beta power, increase of PAF, and steepening of the 35–45 Hz aperiodic slope in the post-stimulus intervals following the “fast” versus “slow” drift rate of moving visual gratings.

### 3.2 Source space analysis

To find cortical sources of the significant effects observed at the sensor level for alpha and beta power, as well as for the 35–45 Hz aperiodic slope, we performed a source localization analysis. Although PAF also demonstrated significant condition- and intensity-related changes, we did not perform this analysis for PAF, because many participants lacked distinct alpha peaks outside the posterior cortical areas.

**Fig. 5.**
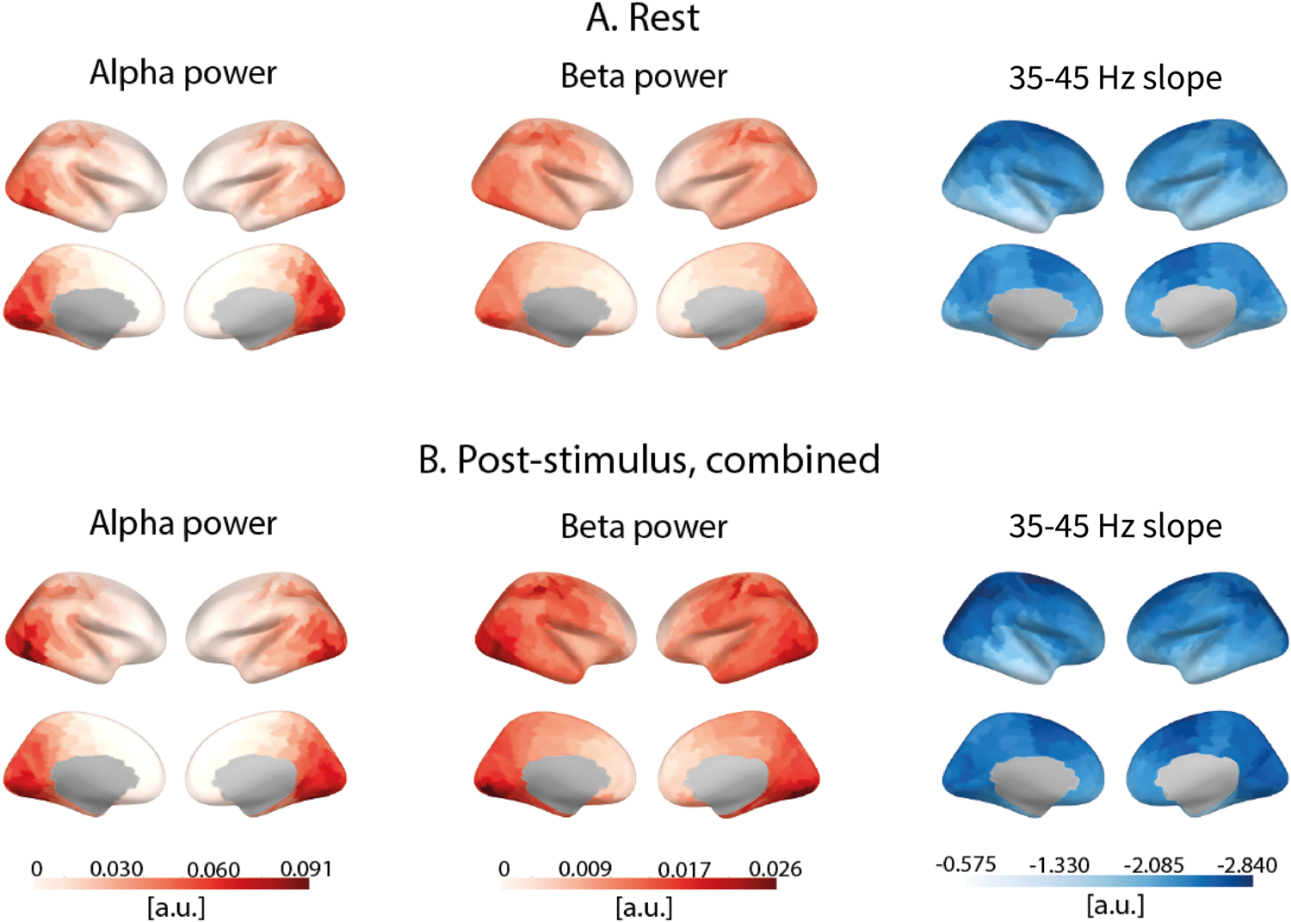
Cortical distribution of periodic alpha power, periodic beta power, and 1/f aperiodic slope estimated in 35–45 Hz range during (A) rest and (B) post-stimulus intervals combined over the three types of the preceding visual stimuli.

**Fig. 6.**
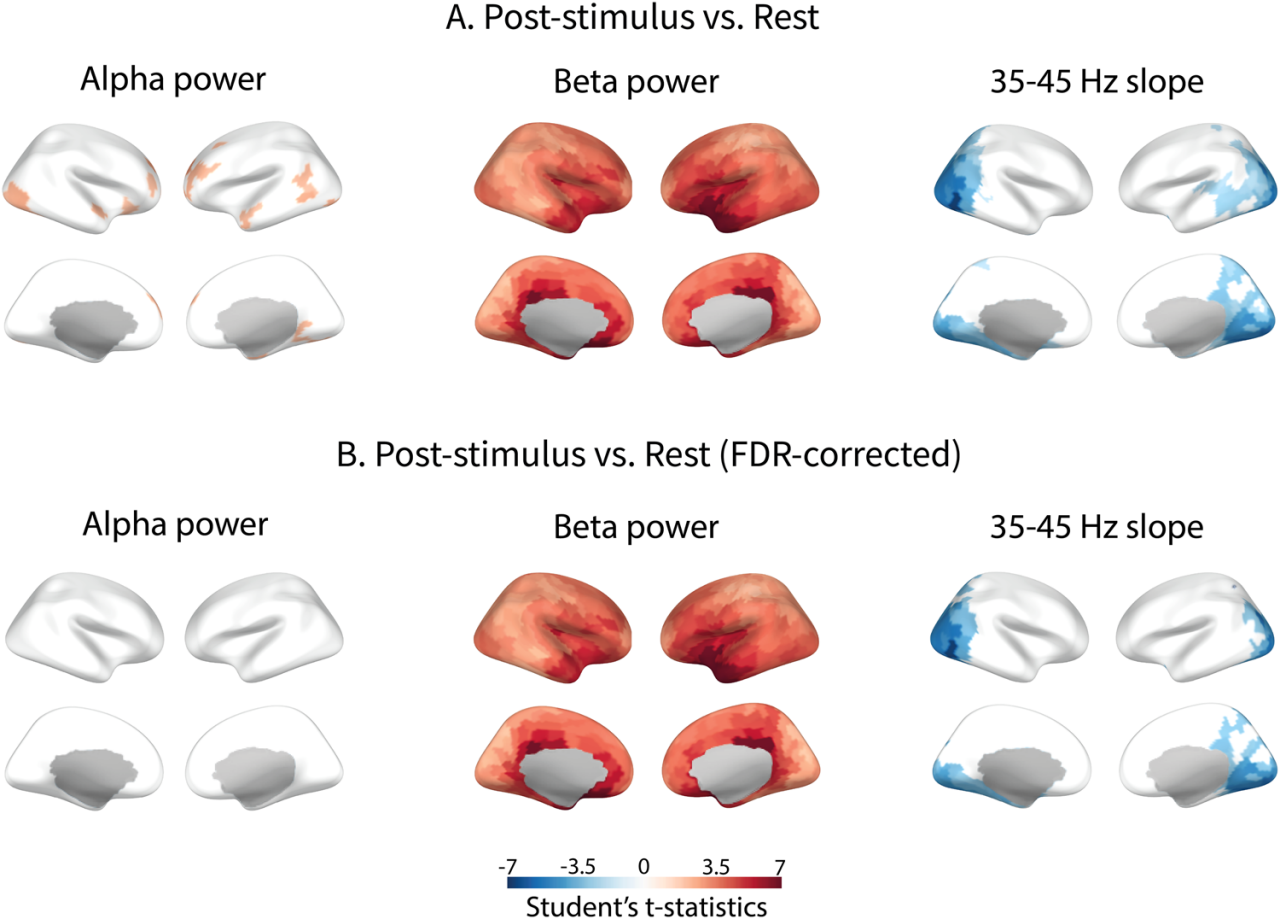
Statistical maps of difference in inhibition-sensitive MEG parameters between rest and post-stimulus intervals, pooled for “after-slow”, “after-medium”, and “after-fast” trials. On the color-coded t-values scale, significant increases and decreases in parameter values from rest to post-stimulus condition are indicated in red and blue, respectively. (A) Differences significant (p < 0.05) without correction for multiple comparisons. (B) Differences significant (p < 0.05) after FDR correction.

Figure 5shows the cortical distribution of alpha and beta periodic power, as well as that of 1/f aperiodic slope at rest and in the post-stimulus period (all drift rates of visual gratings combined).Figure 6shows cortical regions where differences in spectral power and aperiodic slope between rest and post-stimulus conditions were significant.

The decrease in the aperiodic spectral slope from the rest to post-stimulus interval was observed in the posterior areas, including the primary and secondary visual cortex. Unlike that for the slope, differences in the beta power between rest and post-stimulus conditions were widespread and most significant in the cingulate cortex, orbitofrontal cortex, and insula.

**Fig. 7.**
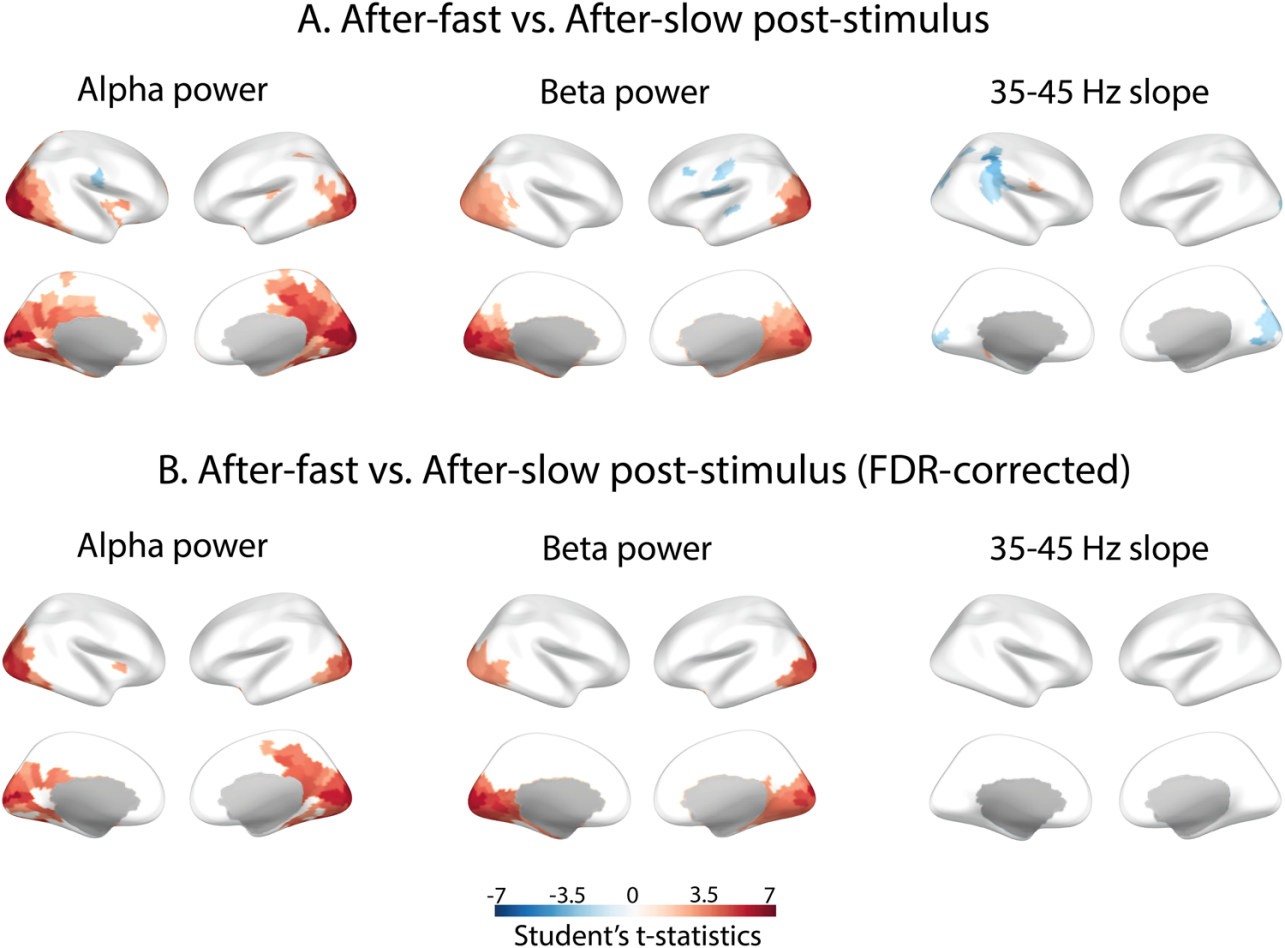
Statistical maps of the differences between “after-slow” (drift rate 1.2 °/s) and “after-fast” (drift rate 6.0 °/s) post-stimulus intervals. On the color-coded t-values scale, significantly higher and lower parameter values in the “after-fast” compared to “after-slow” intervals are indicated in red and blue, respectively. (A) Differences significant (p < 0.05) without correction for multiple comparisons. (B) Differences significant (p < 0.05) after FDR correction.

Figure 7illustrates differences in the MEG parameters between “after-fast” versus “after-slow” conditions. The period after the fast-moving stimulus (more intense stimulation) compared to the period after the slow-moving stimulus (less intense stimulation) was characterized by greater alpha and beta power in visual cortical areas. For alpha power, the difference was also observed in the right precuneus, posterior cingulum, and retrosplenial cortex. The 35–45 Hz aperiodic slope was more negative after more intense stimulation in the right supramarginal area and bilaterally in the visual cortex. Although the stimulation intensity-related changes of the 35–45 Hz aperiodic slope did not survive correction for multiple comparisons at the source level, given the significant results at the sensor level (Fig. 3B), we considered these changes to be reliable.

To sum up, the source-level results showed that, compared to rest, the period after cessation of visual stimulation was characterized by a broad increase in beta power and steepness of the aperiodic slope in visual cortical areas. Increases in alpha and beta power and steepening of the 35–45 Hz aperiodic slope after more intense compared with less intense visual stimulation were observed more locally in the visual cortex and nearby areas.

### 3.3 The link between post-stimulus inhibition and low neurological threshold

We expected that inter-individual differences in post-stimulus inhibition might be related to individual variability in subjective sensory sensitivity and avoidance. To test this prediction, we calculated correlations between subjects’ scores on the Low Neurological Threshold and condition- or intensity-related changes in inhibition-sensitive parameters (PAF, alpha power, beta power, 1/f aperiodic slope) (Table 4). Only those parameters, which demonstrated significant condition- or intensity- related differences at the sensor level were used for this analysis (Fig. 3A,B).

**Table 4.** Pearson correlations between Low Neurological Threshold scores and condition- or intensity-related changes in MEG parameters sensitive to post-stimulus inhibition.^#^

	Post-stimulus intervals vs. rest			“After-fast” vs. “after-slow”			
	PAF, difference	Beta power, % change	35-45 Hz slope, difference	PAF, difference	Alpha power, % change	Beta power, % change	35-45 Hz slope, difference
Low Neurological Threshold	R = 0.44	R = 0.09	R = -0.31	R = -0.24	R = 0.57 *	R = 0.52	R = -0.19

# All the parameters were estimated at the sensor level. Changes in PAF and aperiodic slope were calculated as the absolute difference between conditions, while changes in periodic power were calculated as the relative difference in % (see Methods for details). Three subjects lacking alpha peaks in one or more conditions and one participant lacking data on Low Neurological Threshold were excluded from this analysis, leaving a total of 21 subjects.

*p = 0.042, FDR-corrected.

**Fig. 8.**
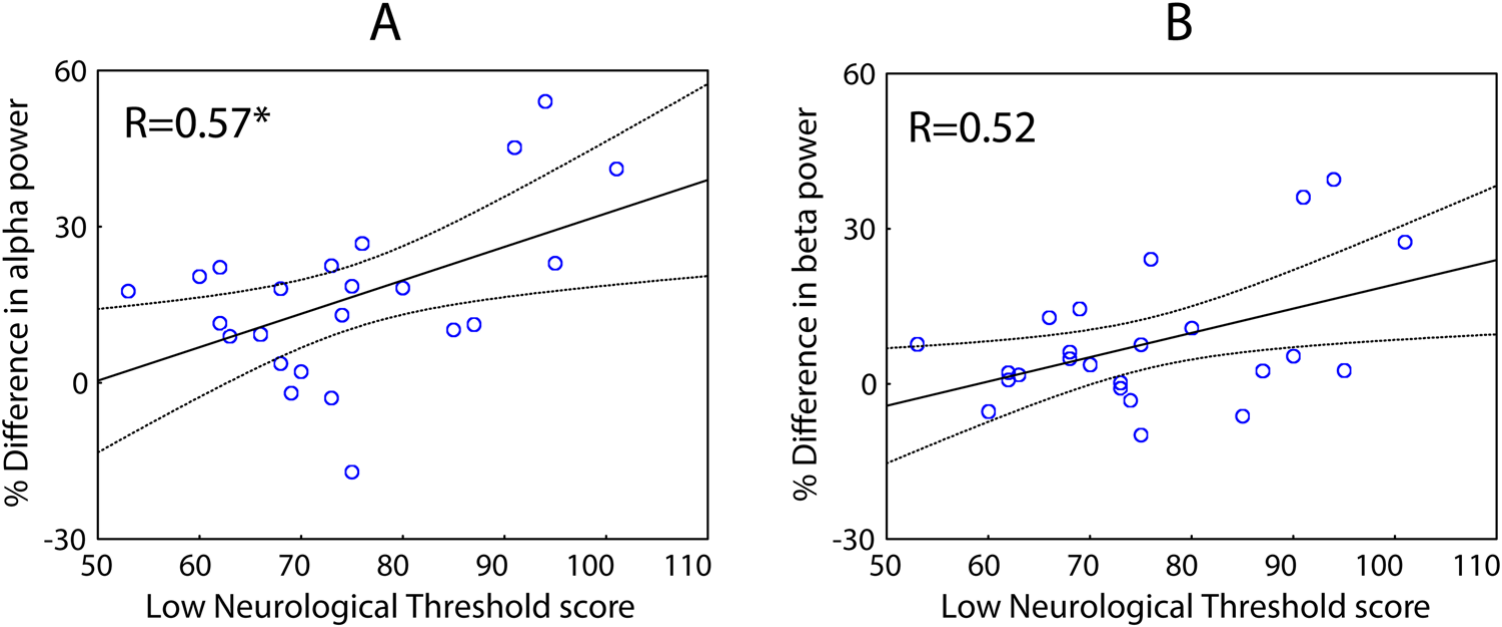
The relationship between intensity-related increase in periodic alpha (A) and beta (B) power from “after-slow” to “after-fast” condition and Low Neurological Threshold (individual sensory sensitivity and avoidance) scores. The relative difference between “after-fast” and “after-slow” post-stimulus intervals is calculated for the sensor space data, as 2*(after-fast–after-slow)/(after-fast + after-slow)*100%.

Higher scores on the Low Neurological Threshold (i.e., greater sensitivity to sensory stimulation and avoidance of it in daily life) correlated with greater intensity-related changes between “after-slow” and “after-fast” conditions in alpha (p = 0.042, FDR-corrected) and—as a tendency—beta (p = 0.06, FDR-corrected) power (Fig. 8A,B).

### 3.4 Behavioral performance in the visual task

Because participants were instructed to respond to the cessation of grating drift by pressing a button, differences in MEG parameters in the visual task could be related not only to different intensities of visual stimulation but also to differences in motor response (reaction time (RT), error rate).

RmANOVA with factors Phase and Condition (“slow”, “medium”, “fast”) has shown the significant main effect of Condition (F(2,48) = 11.3, p = 0.0001) on RT. RT decreased from the “slow” to “medium” and “fast” conditions (mean slow RT = 359 ms, mean medium RT = 346 ms, mean fast RT = 346 ms). Because the omission and commission error rates were not normally distributed, we used nonparametric Friedman ANOVA to analyze the effect of Condition on these variables. For this analysis, we averaged error rates between visits, since no effect of Phase was found for any of these variables (Wilcoxon signed-rank test, all p’s > 0.05, uncorrected for multiple comparisons). There were no differences in omission rate between conditions (ANOVA Chi-Square (N = 25, df = 2) = 1.24, p = 0.53; median “slow” omission = 1.1%, median “medium” omission = 1.1%, median “fast” omission = 0.56%). However, velocity-dependent differences in commission errors were observed: their percentage decreased with increasing velocity (ANOVA Chi Square (N = 25, df = 2) = 10.5, p = 0.005; median “slow” commission = 5.0%, median “medium” commission = 3.5%, median “fast” commission = 2.9%).

Because subjects responded to stopping the fast drifting grating faster and with fewer commission errors than to stopping the slow-drifting grating, to test whether these changes in performance were related to changes in MEG parameters, we performed correlation analyses (Table 5).

**Table 5.** Spearman correlations between condition-related changes in behavioral performance (RT, commission errors) and condition-related changes in MEG parameters assessed at the “sensor level”.

	N	Spearman R	T(N-2)	p-value, uncorrected
RT, % change * and:				
alpha frequency, Fast–Slow	22	-0.26	-1.18	0.252
alpha power, % change	25	0.136	0.65	0.524
beta power, % change	25	-0.04	-0.18	0.858
1/f slope, Fast–Slow	25	0.06	0.31	0.762
% Commission errors, Fast–Slow and:				
alpha frequency, Fast–Slow	22	-0.03	-0.14	0.893
alpha power, % change	25	-0.51	-2.88	0.008 #
beta power, % change	25	-0.35	-1.80	0.085
1/f slope, Fast–Slow	25	0.14	0.70	0.491

*Changes in RT and spectral power were calculated as (Fast - Slow)/Slow*100.

#FDR corrected p-value = 0.064.

Changes in RT from the “slow” to “fast” condition did not correlate with changes in any of the functional inhibition parameters. On the other hand, changes in commission errors (i.e., fast responses made before a stimulus could be processed) tended to correlate with changes in alpha power: a greater decrease in the error rate from the slow to fast condition was related to a greater increase in post-stimulus alpha power.

We next tested whether condition-related changes in the percent of commission errors were associated with individual differences in the “Low Neurological Threshold”. Higher scores on the Low neurological threshold were related to a greater reduction in the error rate from the slow to fast condition (Pearson R (N = 24) = -0.48, p = 0.02).

To summarize, in our experimental paradigm, an increase in gratings’ drift rate was associated with faster (i.e., shorter RT) and less impulsive performance. While the decrease in RT did not correlate with changes in any of the investigated MEG parameters, the decrease in commission errors correlated with a velocity-dependent increase in post-stimulus alpha power. Given that the effects of drift rate on impulsive responses and alpha power were more pronounced in subjects with higher sensory sensitivity/avoidance scores, their common source lies in the regulation of visual processes rather than in motor control factors.

## 4 Discussion

The neuronal studies suggest that cessation of sensory stimulation is followed by an increase in functional inhibition, which exceeds the baseline level and may serve to restore the functional state of the repeatedly stimulated cortical areas. Here, we investigated whether the changes in MEG-based parameters previously associated with inhibition (increases in periodic alpha-beta power, increases in alpha frequency, and steepness of aperiodic 1/f slope) were sensitive to post-stimulus enhancement of inhibition compared to the resting state. We also investigated whether changes in these parameters are proportional to the intensity of the preceding visual stimulation. We then tested whether changes in MEG parameters associated with post-stimulus inhibition correlated with subjective sensory sensitivity and avoidance.

Our main results show that all four investigated parameters depend on visual stimulation intensity. Their intensity-dependent changes concordantly indicate a greater shift to functional inhibition after cessation of more intense versus less intense visual input, and significant correlations between some of these parameters (and their changes) suggest that they are likely to share some common underlying mechanisms.

### 4.1 MEG indices of post-stimulus functional inhibition scale in proportion to the intensity of visual stimulation

We found that all four examined MEG indices were sensitive to the strength of preceding visual stimulation (faster vs. slower drift rate of visual gratings) and, except for the PAF, displayed correlated changes. The small differences in performance RT between stimulation conditions (“slow”: 359 ms, “medium” & “fast”: 346 ms) are unlikely to explain these results, since no correlations were found between changes in MEG parameters and changes in RT (Table 5). Furthermore, while time courses of alpha-beta power in the post-stimulus intervals were very similar in all three experimental conditions, the increase of alpha-beta power was strongest after the most intense stimulation (Fig. 4). Thus, the observed changes in MEG parameters are compatible with an intensity-dependent increase in post-stimulus functional inhibition.

Note, that the term “post-stimulus” here refers to the time window after visual stimulation has ceased. These results support the hypothesis that post-stimulus inhibition increases proportionally to the strength of preceding excitation (Haigh et al., 2015;Mullinger et al., 2017). Whereas associations between the intensity of preceding visual stimulation and increases in alpha and/or beta power in the post-stimulus interval have been reported previously (Mullinger et al., 2017;Stevenson et al., 2011), we showed such an association for PAF and aperiodic 1/f slope for the first time.

Greater intensity of the preceding stimulation was associated with a steeper aperiodic slope in stimulated visual areas (Fig. 3). Since the steepness of the aperiodic slope measured in high-frequency range of the power spectrum (>30 Hz) is associated with an overall decrease in neural excitability (Gao et al., 2017;Wiest et al., 2023), this finding suggests that greater activation of the visual cortex by incoming visual input is followed by a proportionally greater functional inhibition in the post-stimulus interval.

Consistent with the results of Muthukumaraswamy and Liley (Muthukumaraswamy & Liley, 2018), who analyzed resting-state MEG and EEG, the greater negativity of the aperiodic 1/f slope estimated at posterior sensors correlates with greater power of periodic alpha-beta oscillations. Here, this correlation was observed for both resting-state and post-stimulus intervals (Table 2). Moreover, changes in these parameters as a function of stimulation intensity (from “after-slow” to “after-fast” interval) were also correlated: greater negativity (steepness) of the slope was associated with a greater increase in the power of periodic alpha-beta oscillations (Table 3b). Thus, the strength and direction of intensity-related changes in aperiodic 1/f slope and power of periodic alpha-beta oscillations point in the same direction: once the intensity of the visual stimulation is going up, the post-stimulus state of the visual cortex undergoes a shift towards inhibition. This push-pull relationship between the intensity of the visual input and post-stimulus changes in inhibition-sensitive MEG parameters is compatible with the hypothesis that functional inhibition plays a homeostatic role in balancing cortical excitability after intensive stimulation (Mullinger et al., 2017).

Weak but significant correlations between changes in post-stimulus periodic power and aperiodic slope suggest some similarity in the underlying mechanisms. This is not unexpected, since a number of studies relate steeper aperiodic slope and higher alpha-beta power to a state of increased functional inhibition characterized by reduction of neuronal firing (alpha-beta power:Bonnefond & Jensen, 2015;Haegens et al., 2011;Iemi et al., 2022;van Kerkoerle et al., 2014; aperiodic slope:Gao et al., 2017;Wiest et al., 2023).

While, according to modeling, animal and intracranial studies the link between changes in aperiodic slope and neural excitability is straightforward—a steeper slope directly reflects stronger local inhibition (Gao et al., 2017;Wiest et al., 2023), the associations between inhibition and changes in alpha-beta power are more complex. Functionally relevant increase of alpha-beta power is thought to reflect either inhibitory top-down control (Bonnefond & Jensen, 2012;Jensen et al., 2002;Klimesch et al., 1999;Payne et al., 2013), or be secondary to competitive interactions between strongly activated task-relevant cortical regions and task-irrelevant ones, the neuronal activity of which is inhibited to prevent interference (Jensen, 2023;Noonan et al., 2018). At first glance, the latter interpretation is not consistent with the current finding, because the intensity-dependent post-stimulus increase in alpha (beta) power is localized to the task-relevant visual cortical areas. However, post-stimulus alpha-beta synchronization may be a delayed consequence of competitive interactions between populations of neurons in the same cortical area occurring during sensory stimulation (i.e., surround inhibition). For example,Boorman et al. (2015)have found that the regions surrounding the stimulated receptive field in somatosensory cortex in animals demonstrated a negative hemodynamic response that outlasted the “positive hemodynamic response” and correlated with prolonged (300–2000 ms after the termination of the stimulation) suppression of broad-band gamma power (i.e., suppression of neural activity, seeManning et al., 2009). Since large high-contrast moving gratings used in our study activate both neurons’ receptive fields and their suppressive surrounds and induce strong surround inhibition (Gieselmann & Thiele, 2008), alpha-beta synchronization and steepening of the 1/f aperiodic slope that we observed in the post-stimulus interval may reflect such local residual inhibitory processes resulting from competitive interactions*within the same visual area.*This hypothesis is indirectly supported by the results of the iEEG/fMRI study ofHarvey et al. (2013)who showed that surround inhibition in the human primary visual cortex is associated with local increases in alpha activity in the population receptive fields. Presumably, cessation of stimulation can trigger synchronization of these local alpha oscillators over an extensive region of visual cortex, resulting in alpha-beta “rebound” detected by MEG. In this case, the dependence of the post-stimulus alpha-beta increase on the grating drift rate (Fig. 3) can be explained by stronger surround inhibition induced by stimuli drifting at a higher than at a lower rate (seeOrekhova et al., 2020for discussion). Thus, the post-stimulus alpha-beta “rebound” might be a secondary consequence of excitatory-inhibitory interactions driven in the feed-forward way by preceding visual input.

On the other hand, we found some indirect evidence for top-down influences on post-stimulus periodic alpha-beta power. In our participants, subjective hypersensitivity estimated by Low Neurological Threshold score correlated with a relatively*greater*intensity-related enhancement in alpha-beta power in the post-stimulus interval (Fig. 8,Table 4). Notably, this correlation was absent for the aperiodic slope (Table 4). This suggests that some additional factors upregulate post-stimulus alpha-beta power over and above the local residual inhibitory processes. According to the previous literature, periodic alpha power in sensory cortical areas, although correlating with the strength of high-frequency neural response to sensory input, a direct measure of cortical excitability (Iemi et al., 2022), is also modulated by top-down processes, such as, for example, attention engagement/disengagement that are accessible to awareness providing a basis for subjective feeling of stimulation intensity (Kerr et al., 2011;Klimesch, 2012;Peng et al., 2015;Samaha et al., 2020).

It is likely that, similar to people with somatic anxiety (Bouziane et al., 2022), healthy individuals scoring high on the “Low Neurological Threshold” exerted greater top-down inhibitory control over excitability of the exteroceptive sensory (in this case visual) cortical areas than less sensitive individuals. Stronger top-down inhibitory control over the highly excited visual cortex may lead to a proportionally higher increase of alpha-beta power after intensive stimulation and explains its link to subjective sensory sensitivity and avoidance.

These considerations suggest that post-stimulus alpha-beta “rebound” in visual cortex may reflect both feed-forward and feedback inhibitory processes, and evaluation of their relative contributions to this phenomenon deserves further investigation (see, e.g.,Khan et al., 2015).

### 4.2 Dissociation between changes in periodic alpha-beta power and 1/f aperiodic slope from resting state to post-stimulus interval

The contrast between the resting state and post-stimulus period showed that the latter condition was characterized by steepening of the aperiodic slope in the visual cortex (Figs. 3A,6). This finding, along with the increase in periodic beta power and PAF in posterior sensors (Fig. 3A), is consistent with an increase of inhibition during the post-stimulus period compared to rest. Since participants were presented with the same fixation cross in both conditions, these results cannot be explained by differences in visual input but rather reflect internal processes related to the cessation of visual stimulation. Thus, these MEG findings agree with the results of other MEG/EEG studies in humans (Mullinger et al., 2013,^2017^;Stevenson et al., 2011) and neuronal studies in animals (Boorman et al., 2015;Logothetis et al., 2001;Shmuel et al., 2006), in which a post-stimulus enhancement of inhibition above the baseline level was observed.

However, the pattern of differences in MEG parameters between the post-stimulus period and rest cannot be explained solely by post-stimulus increase of inhibition in the stimulated cortical areas. First, cortical topography of condition-related changes was markedly different for aperiodic slope and beta power (Fig. 7). Whereas the difference in the aperiodic slope between rest and post-stimulus interval was limited to the visual cortex and surrounding areas, the differences in beta power were widespread.

One obvious source of increased beta power during the post-stimulus period relative to rest in our study is the post-movement beta rebound (PMBR) associated with the button press that subjects performed in the visual task. Since the PMBR begins about 230 ms after movement termination and lasts several hundred milliseconds (Jurkiewicz et al., 2006), it should coincide with our analysis window (Fig. 1B). However, the maximal amplitude of PMBR is expected in the precentral gyrus, contralateral to the movement side (Gaetz et al., 2010;Jurkiewicz et al., 2006;Wilson et al., 2010). Therefore, the increase of beta power in large-scale cortical networks in the post-stimulus period (Fig. 6) cannot be accounted for solely by PMBR.

Interestingly, the most reliable increase in beta power during the post-stimulus period compared to rest was observed in the cingulate cortex, orbitofrontal cortex, and insula. Since these areas are involved in emotional and autonomic regulation (Ferraro et al., 2022;Hänsel & von Känel, 2008;Stevens et al., 2011), the beta synchronization may be related to the subjective experience of visual stimulation. While moving visual gratings used in our study were not explicitly aversive, subjects often rated them as unpleasant. Therefore, the post-stimulus beta power increase in the ventromedial prefrontal cortex and anterior insula may reflect downregulation of emotional arousal and/or autonomic activity during our “mildly unpleasant” visual experiment. The lack of influence of intensity of the preceding stimulation on post-stimulus beta power in the respective high-order cortical areas (Fig. 6) can be explained by the tonic nature of these regulatory processes: stimuli were presented at random, and the stimulation intensity (drift rate) in the upcoming trial could not be predicted.

Although the mechanisms of the widespread increase in beta oscillations after cessation of intense visual stimulation are beyond the scope of the present work, we can cautiously offer an explanation. The increase in ongoing high-frequency alpha and/or beta power in the ventromedial prefrontal cortex has been previously observed after vigorous physical activity (Hosang et al., 2022) and linked to the enhanced blood level of serotonin (5-HT) (Fumoto et al., 2010). The 5-HT is known to attenuate the brain excitatory state induced by physiological arousal due to its inhibitory effect on cortical neuronal activity through 5-HT1A receptors, which are abundant in the ventromedial prefrontal cortex (Celada et al., 2013). A recent study reported that a stressful event, electrical stimulation of a finger, elicits a post-stimulus increase of MEG beta power in the anterior cingulum that strongly correlates with transient short-latency inhibition of peripheral sympathetic nerve activity, suggesting a common mechanism underlying both responses (Riaz et al., 2022). Given that 5-HT1A receptors are implicated in both sympatho-inhibition (Jonnakuty & Gragnoli, 2008) and downregulation of neuronal activity in ventromedial PFC (Celada et al., 2013), a serotonin-dependent pathway is a possible candidate mechanism for inhibitory control of post-stressful cortical inactivation through beta synchronization. A similar mechanism may work to increase beta power and tonically attenuate the excitatory state associated with unpleasant visual stimulation in our study. Regardless of the exact mechanisms, our results show that reduced excitability of the visual areas does not fully characterize inhibition-related differences in beta power between rest and post-stimulus intervals.

Furthermore, we found no differences in alpha power between rest and post-stimulus conditions. Thus, post-stimulus alpha power in the visual areas is reliably modulated by the intensity of visual stimulation, but not by the very fact of the presence of such stimulation compared to its absence (compareFig. 3Aand3B). This finding is broadly consistent with results ofMullinger et al. (2017), who found little or no changes in alpha power between resting state and intervals following flickering of static visual gratings, but a highly significant increase in alpha power following disappearance of flickering compared to static visual gratings.

A likely explanation of these findings is the high inter-subject variability of physiological arousal in a poorly controlled resting state. Since a low level of physiological arousal is associated with high resting alpha power (Barry et al., 2020;Hutt & Lefebvre, 2022), the transition from a resting state to a more active state during a visual task may lead to a decrease in alpha power not only during visual stimulation, but also in the inter-stimulus intervals. Indeed, we found that this was the case in our participants with higher-than-average alpha power at rest. As the increase in the tonic arousal and post-stimulus inhibition counteract each other, their cumulative effect may result in the absence of difference in alpha power between rest and post-stimulus condition that we observed at the group level.

To sum up, the contrast between the time intervals following cessation of intensive visual stimulation and the resting state revealed partly dissimilar changes in the studied MEG parameters, which could not be explained exclusively by post-stimulus functional inhibition of the stimulated visual areas. The discrepancy between changes in different inhibition-sensitive MEG parameters may be explained by differences in vigilance/tonic arousal between post-stimulus condition and the resting state.

### 4.3 Peak alpha frequency does not correlate with other MEG parameters sensitive to inhibition and changes during menstrual cycle

According to interpretation proposed byBabu Henry Samuel et al. (2018), the post-stimulus and intensity-related increase in PAF (Fig. 2) suggest increased inhibition. Thus, the changes in PAF were in line with changes in other MEG parameters studied (alpha-beta power, aperiodic slope). However, changes in PAF did not correlate with changes in other “inhibition-based” MEG parameters, suggesting that PAF provides distinct information on post-stimulus functional inhibition (Tables 3A,B).

The lack of correlations between PAF and alpha power agrees with the previous literature, which shows that, depending on the experimental condition, an increase in alpha frequency may be accompanied by either decrease (Haegens et al., 2014) (for review seeMierau et al., 2017) or increase (Wianda & Ross, 2019; the present study) in alpha power. It is also in line with our observation that only PAF was modulated by the phase of the MC. Regardless of the experimental condition, PAF was higher in the luteal phase than in the follicular phase. The same differences in the alpha frequency between luteal and follicular phases during resting state have been previously described in several studies (Bazanova et al., 2014;Brötzner et al., 2014;Haraguchi et al., 2021). In general, functional dissociation between frequency and power of alpha oscillations is inconsistent with unitary explanation of changes in alpha frequency and power through the single mechanism.

An interesting parallel between PAF and gamma oscillations frequency arises here. Experimental research (Haegens et al., 2011;Mann & Mody, 2010;Walker & Semyanov, 2008) and some modeling studies (Vierling-Claassen et al., 2010) posit that frequency of both gamma and alpha oscillations is determined by the pace of rhythmic inhibition of neuronal spiking, which in turn depends on excitability of inhibitory neurons, albeit of different types (Vierling-Claassen et al., 2010). Indeed, modulation of the tonic excitability of inhibitory interneurons in mice dramatically affects the frequency (but not amplitude) of gamma oscillations (Mann & Mody, 2010). Continuing this analogy, animal studies have shown that natural (e.g., during pregnancy) or experimental changes in steroid hormone concentrations alter gamma frequency, most likely through complex modulation of tonic excitability of inhibitory neurons (Ferando & Mody, 2013). There is an intriguing possibility that MC phase-related change in PAF and correlation of PAF with progesterone concentration (Bazanova et al., 2014) are also caused by modulation of inhibitory neurons’ excitability by steroid hormones. Since excitability of inhibitory neurons is only one of many factors regulating E/I balance, this may explain the concordant (indicating increased post-stimulus functional inhibition with increasing strength of prior input) but uncorrelated changes in PAF and other MEG parameters that, unlike PAF, index the resulting changes in E/I ratio.

Overall, our results suggest the mechanisms mediating changes in PAF are different from those underlying changes in alpha-beta power and aperiodic slope. Although the current explanation for these mechanisms is speculative, the functional dissociation we found between PAF and other putative measures of E/I ratio deserves further investigation because of its physiological relevance and potential implications for the use of MEG/EEG indexes of functional inhibition in translational studies.

## 5 Limitations

Our study has several limitations. First, the short 1-s post-stimulus interval we used (0.2 s–1.2 s after stimulus cessation) may have missed a portion of the post-stimulus inhibitory response, which in MEG/EEG may last several seconds after stimulation stopped (Mullinger et al., 2017;Stevenson et al., 2011). Longer inter-stimulus intervals would allow a more detailed analysis of the post-stimulus changes in inhibition. Second, our study included a relatively small sample of healthy adult women of reproductive age. A study on men is warranted before our findings can be extended to the general population. Another limitation is the use of a single power law model to separate between periodic (alpha and beta) and aperiodic activity. Due to nonlinearity of a relationship between frequency and power of aperiodic neural activity in the log-log space, a “knee” around 15 Hz might be present in our MEG data separating two different 1/f regimes, each with their own exponent (Ibarra Chaoul & Siegel, 2021). However, since the goodness of fit of the FOOOF model in the alpha-beta range did not vary significantly as a function of the experimental condition, we hope that this approach does not systematically affect the estimation of condition-related changes in periodic activity. Finally, our experimental task required manual response, which could potentially affect the post-stimulus interval in this study. Although our analysis suggests that this factor does not significantly affect the results and conclusions of our study, a study that does not include manual response would need to be conducted to confirm this.

## 6 Conclusions

Our results confirm the previous finding linking the “overshoot” of alpha-beta power following cessation of visual stimulation to functional inhibition and extend them by showing that the aperiodic 1/f slope estimated in the high-frequency range (35–45 Hz) and alpha peak frequency also reflect a shift toward inhibition in the post-stimulus activity of the human visual cortex. The concordant changes in all inhibition-sensitive parameters associated with changes in stimulation intensity are consistent with the idea that post-stimulus inhibition is proportional to the intensity of the preceding visual stimulation. Whereas changes in the 35–45 Hz aperiodic slope from resting to post-stimulus period clearly indicate enhanced inhibition in the stimulated visual cortex, the concurrent modulations of periodic alpha and beta power are more complex and presumably reflect processes not limited to the downregulation of the E/I ratio in stimulated sensory cortical areas.

## Supplementary Material

Supplementary Material

Supplementary Materials_HTMLs

## Data Availability

The data and code used to derive the results of this study are available upon request from the corresponding author.
