# Supplementary material for: Changes in high-frequency aperiodic 1/f slope and periodic activity reflect post-stimulus functional inhibition in the visual cortex
Source: Imaging Neurosci (Camb). 2024 Apr 26;2:imag-2-00146. doi: 10.1162/imag_a_00146 (PMC12247559; doi:10.1162/imag_a_00146)
Supplement: Supplementary Material [file imag_a_00146-supp.pdf]

Changes in high-frequency aperiodic 1/f slope and periodic activity reflect post-stimulus functional inhibition in the visual cortex.

Running title: 'Post-stimulus inhibition in the visual cortex'

Viktoriya O. Manyukhina[1,2], Andrey O. Prokofyev[1], Tatiana S. Obukhova[1], Tatiana A. Stroganova[1], Elena V. Orekhova[1]*

[1] Center for Neurocognitive Research (MEG Center), Moscow State University of Psychology and Education, Moscow, Russian Federation

[2] National Research University Higher School of Economics, Moscow, Russian Federation

*Corresponding author (Orekhova.elena.v@gmail.com)

Supplementary materials

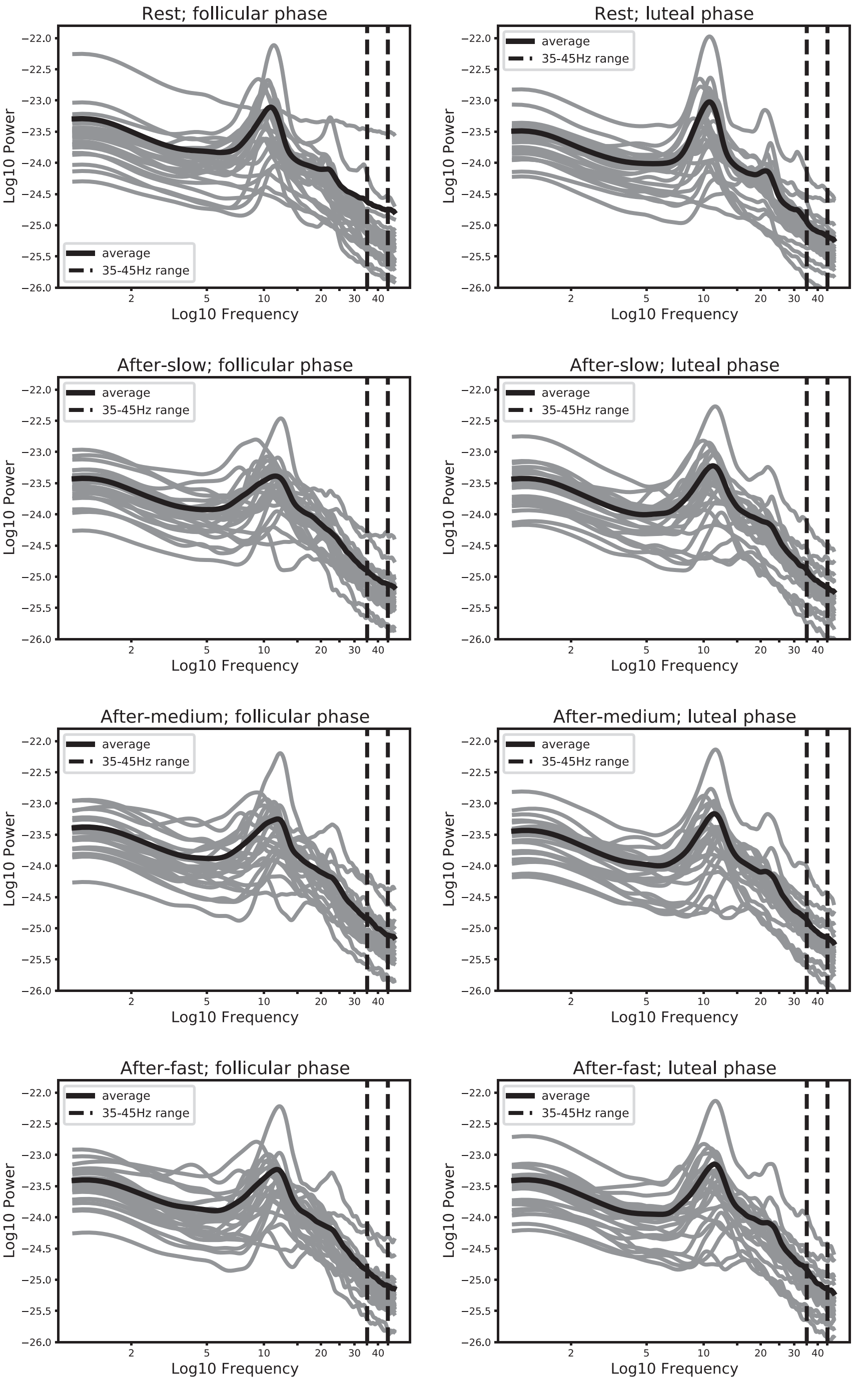

Figure S1. Power spectra averaged for a selection of nine posterior pairs of gradiometers. Spectra are shown separately for each visit (during the follicular or luteal phase of the menstrual cycle) and for each condition (rest and intervals after cessation of visual stimulation with gratings drifting at slow, medium or fast speeds). Individual spectra are shown in grey, the mean spectrum in black. Dashed lines mark 35-45 Hz interval.

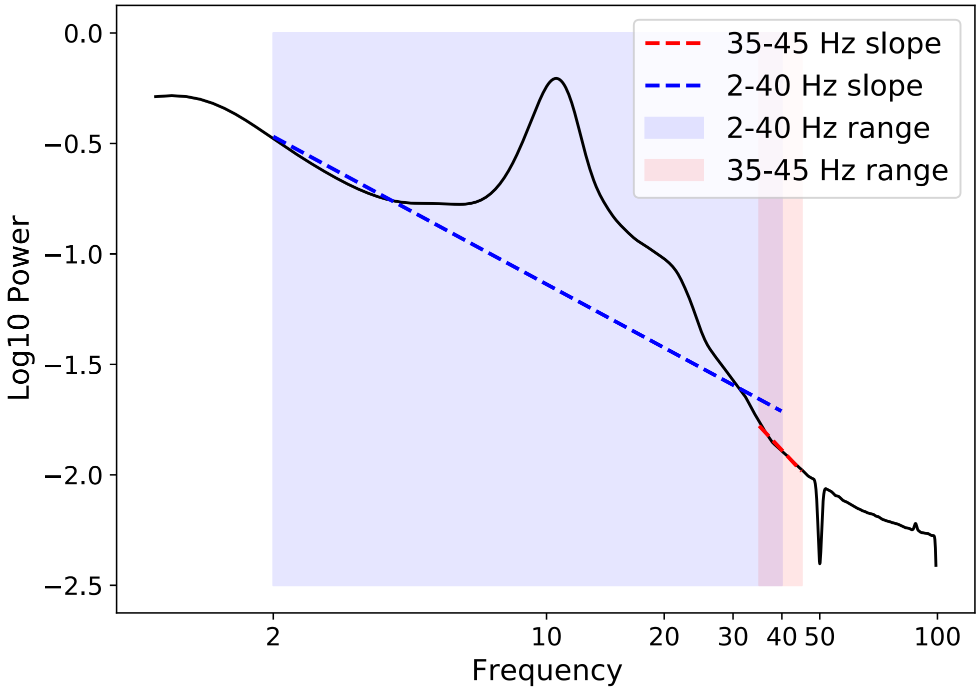

Figure S2. Illustration of aperiodic spectral slopes estimated for the post-stimulus condition using a linear approximation of the log-log spectrum in the 35-45 Hz range (red dashed line) or FOOOF in the 2-40 Hz range (blue dashed line). Note that the nonlinear drop in spectral power at high frequencies (~>50 Hz) is most likely due to a decrease in SNR due to contamination by ambient noise (see Ibarra Chaoul and Siegel, 2021).

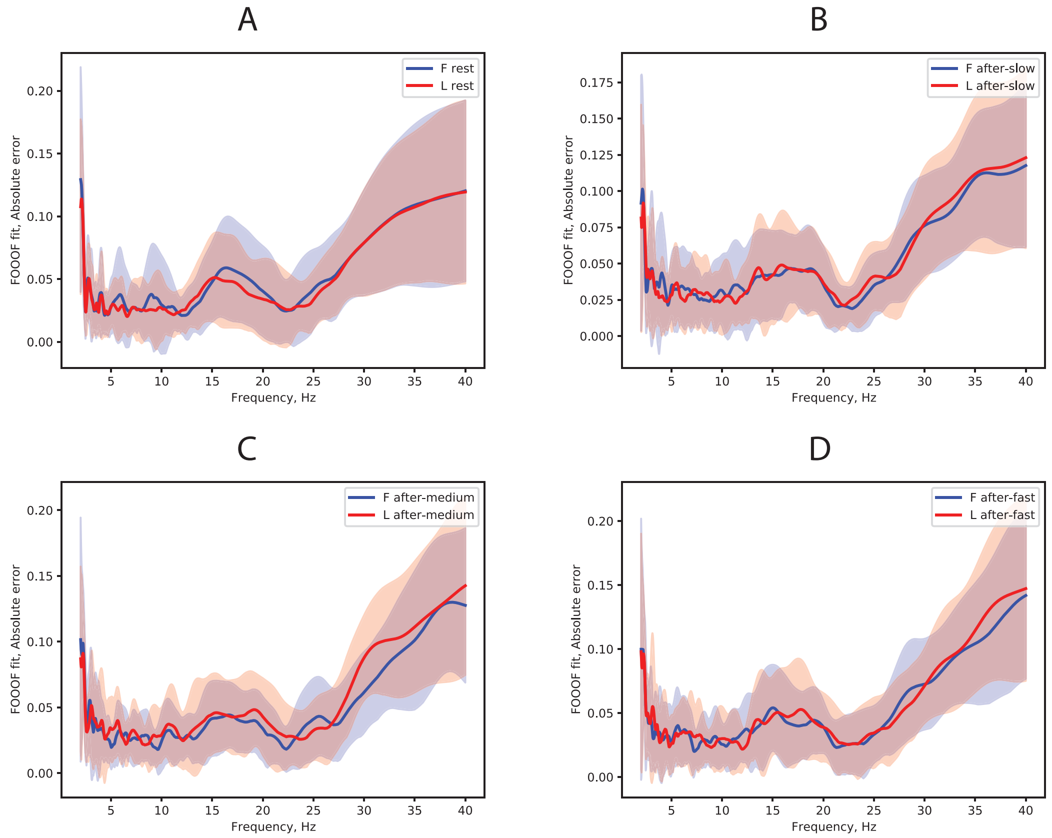

Figure S3. Group average mean absolute frequency-by-frequency error of fit for the full FOOOF model, for the rest (A) and post-stimulus intervals (B, C, and D). Blue color: follicular phase, red: luteal phase. The shading indicates the standard deviation of the error. Note the uneven distribution of errors, which are highest at low (2 Hz) and high (>25 Hz) frequencies.

Results of the analysis of the 35-45 Hz aperiodic slope at the marginal sensors

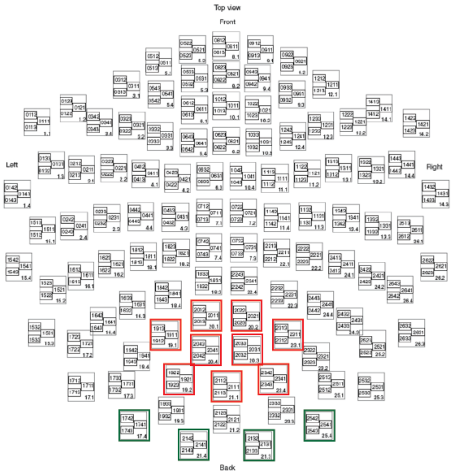

Figure S4. Sensors used for analysis in the 'sensor space'. Each colored square contains 3 sensors, of which only gradiometers (2 sensors in each location) were used for analysis. Sensors marked in green were used to test the putative contribution of muscle artifacts to condition-related differences in 35-45 Hz slope found for the selection of gradiometers marked in red.

To test if the condition- and intensity-related differences in the 35-45 Hz aperiodic slope at the occipital selection of gradiometers (Fig. S4, red squares) can be explained by differences in muscle artifacts, we repeated the analyses for selection of marginal posterior gradiometers closest to the neck muscles (Fig. S4, green squares).

For this selection, the rmANOVA with factors Condition (rest, post-stimulus) and Phase (follicular, luteal) revealed no significant effects of Condition ($F(1,24)=3.6$, $p=0.07$) or Condition x Phase interaction ($F(1,24)=0.01$, $p=0.90$).

Similarly, rmANOVA with factors Intensity ('after-slow', 'after-medium', 'after-fast') and Phase (follicular, luteal) revealed no significant effects of Intensity ($F(2,48)=1.55$, G-G epsilon = 0.96, $p=0.22$) or Intensity x Phase interaction ($F(2,48)=0.07$, G-G epsilon = 0.99, $p=0.93$).

The absence of significant effects of Condition and Intensity for the edge gradiometers indicates that the significant effects found for the selection of 9 pairs of posterior gradiometers (Fig. S4, red squares) are unlikely to be explained by muscle artifacts.

References

Ibarra Chaoul, A., & Siegel, M. (2021). Cortical correlation structure of aperiodic neuronal population activity. *Neuroimage, 245*, 118672. doi:10.1016/j.neuroimage.2021.118672

Muthukumaraswamy, S. D., & Liley, D. T. (2018). 1/f electrophysiological spectra in resting and drug-induced states can be explained by the dynamics of multiple oscillatory relaxation processes. *Neuroimage, 179*, 582-595. doi:10.1016/j.neuroimage.2018.06.068
