# Supplementary material for: Changes in high-frequency aperiodic 1/f slope and periodic activity reflect post-stimulus functional inhibition in the visual cortex
Source: Imaging Neurosci (Camb). 2024 Apr 26;2:imag-2-00146. doi: 10.1162/imag_a_00146 (PMC12247559; doi:10.1162/imag_a_00146)
Supplement: Supplementary Materials_HTMLs [file imag_a_00146-supp-htmls.zip › Supplementary_materials2_HTMLs/Fast_Follicular_spectrum_fit_and_freqbyfreq_error_in_18occip_grads.html]

Filter by tags

- Select all
- ---
- 18-occipital-gradiometers

##### Table of contents

S01, Models fit
S01, Freq-by-freq
S02, Models fit
S02, Freq-by-freq
S03, Models fit
S03, Freq-by-freq
S04, Models fit
S04, Freq-by-freq
S05, Models fit
S05, Freq-by-freq
S06, Models fit
S06, Freq-by-freq
S07, Models fit
S07, Freq-by-freq
S08, Models fit
S08, Freq-by-freq
S09, Models fit
S09, Freq-by-freq
S10, Models fit
S10, Freq-by-freq
S11, Models fit
S11, Freq-by-freq
S12, Models fit
S12, Freq-by-freq
S13, Models fit
S13, Freq-by-freq
S14, Models fit
S14, Freq-by-freq
S15, Models fit
S15, Freq-by-freq
S16, Models fit
S16, Freq-by-freq
S17, Models fit
S17, Freq-by-freq
S18, Models fit
S18, Freq-by-freq
S19, Models fit
S19, Freq-by-freq
S20, Models fit
S20, Freq-by-freq
S21, Models fit
S21, Freq-by-freq
S22, Models fit
S22, Freq-by-freq
S23, Models fit
S23, Freq-by-freq
S24, Models fit
S24, Freq-by-freq
S25, Models fit
S25, Freq-by-freq

S01, Models fit

18-occipital-gradiometers

S01, Freq-by-freq

18-occipital-gradiometers

S02, Models fit

18-occipital-gradiometers

S02, Freq-by-freq

18-occipital-gradiometers

S03, Models fit

18-occipital-gradiometers

S03, Freq-by-freq

18-occipital-gradiometers

S04, Models fit

18-occipital-gradiometers

S04, Freq-by-freq

18-occipital-gradiometers

S05, Models fit

18-occipital-gradiometers

S05, Freq-by-freq

18-occipital-gradiometers

S06, Models fit

18-occipital-gradiometers

S06, Freq-by-freq

18-occipital-gradiometers

S07, Models fit

18-occipital-gradiometers

S07, Freq-by-freq

18-occipital-gradiometers

S08, Models fit

18-occipital-gradiometers

S08, Freq-by-freq

18-occipital-gradiometers

S09, Models fit

18-occipital-gradiometers

S09, Freq-by-freq

18-occipital-gradiometers

S10, Models fit

18-occipital-gradiometers

S10, Freq-by-freq

18-occipital-gradiometers

S11, Models fit

18-occipital-gradiometers

S11, Freq-by-freq

18-occipital-gradiometers

S12, Models fit

18-occipital-gradiometers

S12, Freq-by-freq

18-occipital-gradiometers

S13, Models fit

18-occipital-gradiometers

S13, Freq-by-freq

18-occipital-gradiometers

S14, Models fit

18-occipital-gradiometers

S14, Freq-by-freq

18-occipital-gradiometers

S15, Models fit

18-occipital-gradiometers

S15, Freq-by-freq

18-occipital-gradiometers

S16, Models fit

18-occipital-gradiometers

S16, Freq-by-freq

18-occipital-gradiometers

S17, Models fit

18-occipital-gradiometers

S17, Freq-by-freq

18-occipital-gradiometers

S18, Models fit

18-occipital-gradiometers

S18, Freq-by-freq

18-occipital-gradiometers

S19, Models fit

18-occipital-gradiometers

S19, Freq-by-freq

18-occipital-gradiometers

S20, Models fit

18-occipital-gradiometers

S20, Freq-by-freq

18-occipital-gradiometers

S21, Models fit

18-occipital-gradiometers

S21, Freq-by-freq

18-occipital-gradiometers

S22, Models fit

18-occipital-gradiometers

S22, Freq-by-freq

18-occipital-gradiometers

S23, Models fit

18-occipital-gradiometers

S23, Freq-by-freq

18-occipital-gradiometers

S24, Models fit

18-occipital-gradiometers

S24, Freq-by-freq

18-occipital-gradiometers

S25, Models fit

18-occipital-gradiometers

S25, Freq-by-freq

18-occipital-gradiometers

Created on января 28, 2024 via MNE-Python 0.24.1
